# Young People, Adult Worries: Randomized Controlled Trial and Feasibility Study of the Internet-Based Self-Support Method “Feel the ViBe” for Adolescents and Young Adults Exposed to Family Violence

**DOI:** 10.2196/jmir.6004

**Published:** 2017-06-12

**Authors:** Karin van Rosmalen-Nooijens, Sylvie Lo Fo Wong, Judith Prins, Toine Lagro-Janssen

**Affiliations:** ^1^ Gender & Women’s Health Department of Primary and Community Care Radboud university medical center Nijmegen Netherlands; ^2^ Department of Medical Psychology Radboud university medical center Nijmegen Netherlands

**Keywords:** domestic violence, child abuse, exposure to violence, adolescent, young adult, telemedicine, peer group, peer influence, Internet, feasibility studies, randomized controlled trial, delivery of health care

## Abstract

**Background:**

Adolescents and young adults (AYAs) are of special interest in a group of children exposed to family violence (FV). Past-year prevalence of exposure to FV is known to be highest in AYAs and has severe consequences. Peer support is an effective approach to behavior change and the Internet is considered suitable as a mode of delivery.

**Objective:**

The study aimed to evaluate both effectiveness and feasibility of a randomized controlled trial (RCT) and feasibility study of the Internet-based self-support method “Feel the ViBe” (FtV) using mixed-methods approach to fully understand the strengths and weaknesses of a new intervention.

**Methods:**

AYAs aged 12-25 years and exposed to FV were randomized in an intervention group (access to FtV + usual care) and a control group (minimally enhanced usual care) after they self-registered themselves. From June 2012 to July 2014, participants completed the Impact of Event Scale (IES) and Depression (DEP) and Anxiety (ANX) subscales of the Symptom CheckList-90-R (SCL-90) every 6 weeks. The Web Evaluation Questionnaire was completed after 12 weeks. Quantitative usage data were collected using Google analytics and content management system (CMS) logs and data files. A univariate analysis of variance (UNIANOVA) and mixed model analysis (intention-to-treat [ITT], complete case) were used to compare groups. Pre-post *t* tests were used to find within-group effects. Feasibility measures structurally address the findings. The CONsolidated Standards Of Reporting Trials of Electronic and Mobile HEalth Applications and onLine TeleHealth (CONSORT-EHEALTH) checklist was closely followed.

**Results:**

In total, 31 out of 46 participants in the intervention group and 26 out of 47 participants in the control group started FtV. Seventeen participants (intervention: n=8, control: n=9) completed all questionnaires. Mixed model analysis showed significant differences between groups on the SCL-90 DEP (*P*=.04) and ANX (*P*=.049) subscales between 6 and 12 weeks after participation started. UNIANOVA showed no significant differences. Pre-post paired sample *t* tests showed significant improvements after 12 weeks for the SCL-90 DEP (*P*=.03) and ANX (*P*=.046) subscales. Reported mean Web-based time per week was 2.83 with a session time of 36 min. FtV was rated a mean 7.47 (1-10 Likert scale) with a helpfulness score of 3.16 (1-5 Likert scale). All participants felt safe. Two-thirds of the intervention participants started regular health care.

**Conclusions:**

No changes on the IES were found. SCL-90 DEP and ANX showed promising results; however, the calculated sample size was not reached (n=18). FtV functions best as a first step for adolescents and young adults in an early stage of change. FtV can be easily implemented without extensive resources and fits best in the field of public health care or national governmental care.

**Trial Registration:**

Netherlands National Trial Register (NTR): NTR3692; http://www.trialregister.nl/trialreg/admin/ rctview.asp?TC=3692 (Archived by WebCite at http://www.webcitation.org/6qIeKyjA4)

## Introduction

Family violence (FV) mostly affects women and children—about 30% of all women in a relationship reported to have experienced some form of violence in their relationship [1,2]; and in approximately 60% of the cases, children are living in these violent households [3,4]. Nowadays, it is widely acknowledged that children in violent homes are almost always exposed to this violence. The most common form of exposure to FV is exposure to intimate partner violence, including assaults by parents on siblings or among siblings. There are many possible forms of exposure that vary from direct exposure (seeing or hearing the violence) to indirect exposure (having to deal with the consequences of the violence for daily life) [5]. Recent studies show that 8-12% of all children were exposed to some form of FV in the preceding year [4,6,7]. In the last two decades, numerous studies were published on the effects of exposure to FV on children. Exposure to violence is associated with emotional, behavioral, and adjustment problems as internalizing and externalizing behavior, educational dropout, and mental health disorders such as post-traumatic stress disorder (PTSD), affective and depressive disorders, and suicidal attempts. It is associated with adolescent dating violence, high-risk sexual behavior, teenage pregnancy, and intergenerational transmission—becoming a victim or perpetrator of FV in adult life [8-20]. These consequences closely resemble those of direct victims of physical abuse. In the Netherlands, exposure to FV is therefore considered a form of child abuse [21].

Adolescents and young adults (AYAs) are a group of special interest in a group of children exposed to FV. Hamby (2011) showed that the past-year prevalence of any exposure to FV was highest in 14-17 year olds (13.8%) [6]. Adolescence is an important and life-altering period in human life with tremendous physical and psychological changes [22,23]. In this period, peers are highly important and are considered, more than family, significant others when facing problems.

The transtheoretical stages-of-change model by Prochaska and DiClemente [24] describes the process of intentional behavior change and can be used to describe the situation of AYAs exposed to FV. We hypothesize that AYAs exposed to FV to be in a precontemplation phase. In this phase, they are not yet ready to take action to change their situation. AYAs in a precontemplation phase may be unaware of the abnormality of their living situation and therefore unable to change it. In this phase, education focusing on what is and what is not normal in families could help move AYAs from precontemplation to a contemplation phase. This could make AYAs aware of the abnormality of their home situation and thus help them move from precontemplation to contemplation.

In the contemplation phase, AYAs exposed to FV are aware of the abnormality of this violence but are not committed to change this: they might be hesitant to share experiences with peers or significant others out of shame or fear that their home situation may harm their status (peer pressure) [25,26]. Besides, knowing that AYAs often support their mother by trying to protect her instead of being protected, they are not likely to seek help for themselves [27-29]. To induce change to a preparation phase, in which someone is ready to initiate change, it is needed to educate a person in a general and nonprovocative manner. Therefore, it is important to identify and reach AYAs exposed to FV as soon as possible.

Several reviews have addressed adolescent help-seeking behavior for mental health problems, including being a victim of violence. Barriers include attitude toward health care; confidentiality and trust issues; fear or stress about help-seeking; and lack of knowledge, accessibility, or recognition by others [30-32]. Having detected them, it is challenging to provide health care that is appropriate to both the age group and their specific problems [32-34]. Traditional programs are often not ready for participants in a precontemplation phase—directed at immediate change, they are not suitable for participants who are still weighing the pros and cons of changing their situation. Besides, most interventions need parent involvement, are regional, or do not offer specialized care.

In need of alternative ways to provide low threshold care to AYAs exposed to FV, the Internet could be an effective way to deliver care. In 2013, 100% of the adolescents between 12-25 years old in the Netherlands had access to the Internet [35]. The Internet is used by AYAs to retrieve health information and to self-disclose problems to peers on the Web, rather than asking for help elsewhere [36-38]. eHealth is a rapidly developing and upcoming mode of therapy. Internet-based intervention have been shown to be cost-effective in different health problems [39-43], including mental health in an adolescent population [44-46]. Provision of information, sharing experiences, and peer modeling are known to increase self-management [47-49] and peer- and social support are effective methods to change behavior, both offline and on the Web [49-55]. Furthermore, social support has proven to be effective in adults exposed to violence and is associated with good mental and physical health outcomes [56-58].

It is likely that AYAs exposed to FV will search for information on the Internet too. Therefore, an Internet-based self-help intervention targeted at this group could be a way to reduce barriers to help-seeking and help AYAs exposed to FV to become ready for change. To the best of our knowledge, there are no eHealth interventions for AYAs exposed to FV. Therefore in 2011, “Feel the ViBe” (feel the violence beaten) was developed as an alternative way to reach and support AYAs exposed to FV.

This study first describes a randomized controlled trial (RCT) and then, a feasibility study performed with the following research question: “Is Feel the ViBe an effective and feasible way of reaching and delivering support to adolescents and young adults exposed to family violence?”

## Methods

### Intervention

Feel the ViBe (FtV) is a freely-available, Internet-based self-support method for AYAs exposed to FV (self-assessed) [59,60] with three main goals: (1) providing information, (2) offering (peer) support, and (3) lowering the threshold to regular health care services by supporting participants to move to a higher level of change and to find health care fitting their needs. The intervention comprises a variety of elements, being among others a forum, a chat function, informational pages, and an “ask the expert” function (Table 1). A community manager (CM) moderates the intervention, answers questions, assesses safety, and supports participants when needed, both on demand when asked for, and actively when they judge a participant could use support of additional information. The CM is a semiprofessional with a background in health care and additional training on FV.

eHealth interventions often suffer from the law of attrition: the phenomenon of participants stopping usage or being lost to follow-up [61,62]. To facilitate exposure, we used expert literature and interviews with AYAs exposed to FV to compose the intervention and information on the Web, performed a search-engine optimization (SEO), and included general information about FV on the website of FtV to facilitate exposure for first-time visitors.

Participants could access FtV from any computer; they needed only their login name and password. FtV is to be used ad libitum without endpoint to the intervention; however, to facilitate exposure for returning visitors we included structured events and reminders [63-65]. FtV is described in detail in the study protocol of FtV [60]. The CONSORT-EHEALTH checklist was closely followed [66].

### Participants

Participants included were AYAs aged 12-25 years, exposed to FV as defined previously, and who registered themselves at the homepage of FtV (feel-the-vibe.nl). Participants were included from June 2012 to January 2014. We did not actively recruit participants; all participants found FtV on Google or through other websites. In this early phase, inquiring participants about the type and severity of the violence would enlarge the threshold for participation. Therefore, we chose to consider every potential participant eligible as target group. All participants who started to use FtV were exposed to FV, including 2 participants who were excluded after randomization because of their age. Excluded were participants not in command of the Dutch language, as FtV is in Dutch.

**Figure 1:**
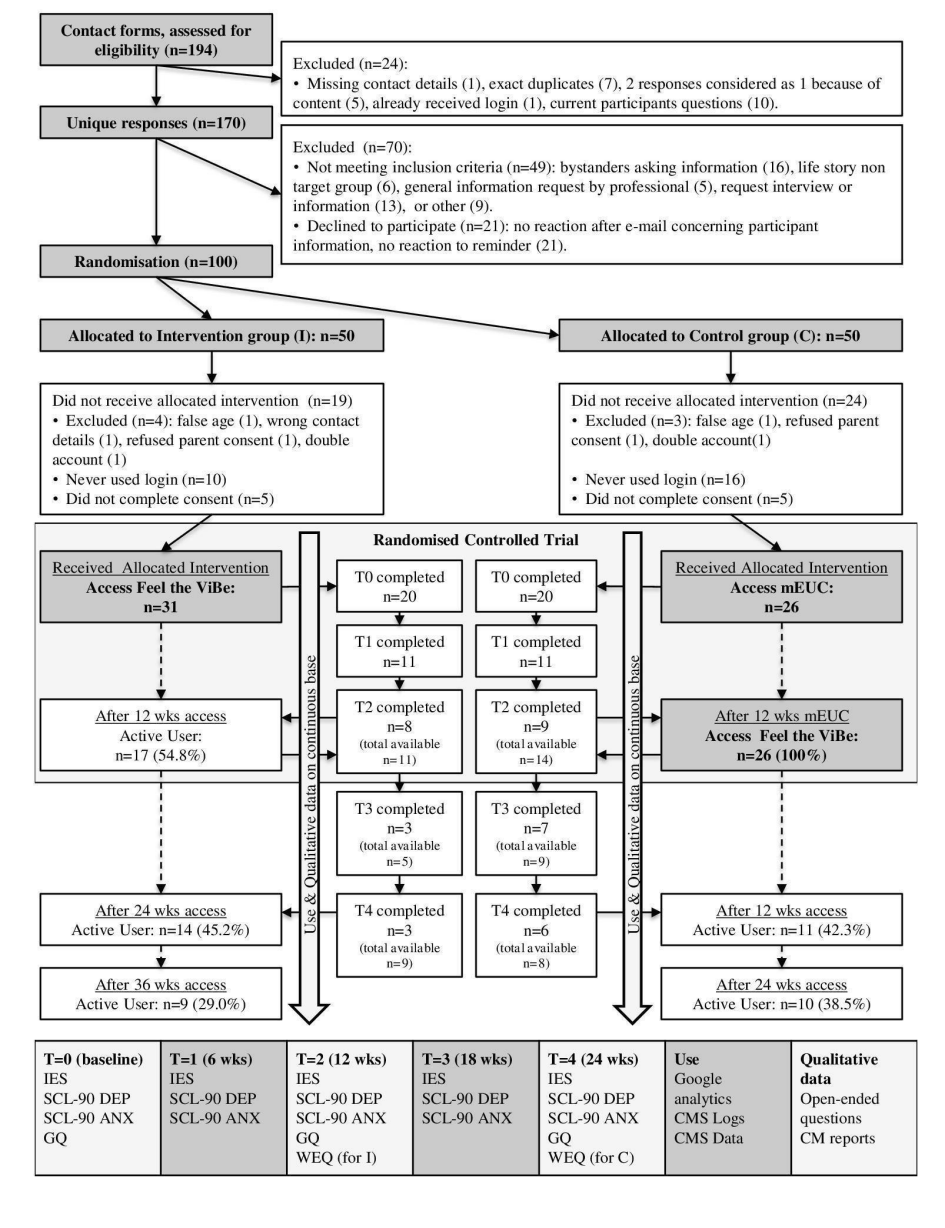
Flowchart.

If participants read the patient information letter available on the homepage and had no further questions, they were eligible to participate and were randomized in 2 parallel groups with a 1:1 allocation ratio: an intervention group, having access to FtV + usual care (UC), and a control group, having access to minimally enhanced usual care (mEUC), meaning that they were placed on a waiting list for 12 weeks with access to 24 h emergency care. Both groups have been extensively described in the study protocol of FtV (Figure 1) [60].

On their first login, participants and parents of participants aged 12 to 16 years gave informed consent electronically. Multiple identities were prevented by making the registration a manual process, including an Internet protocol (IP) address check and email contact, and the informed consent process.

A safety protocol ensured participant safety: FtV is based on a secured server and participants had to use a nickname. All personal data on the Web was removed by a CM, who also monitored the intervention. Participants could contact the CM in case of emergency, independent of their randomization group and electronic consent. All participants were obligated to give contact details of any adult they trust. In case of severe danger, the CM could contact this person with consent of the participant; or, if a participant was below 16 years, the CM could also contact without consent. Participants could not be blinded due to the nature of the study. Participants, recruitment, randomization, consent, and intervention are all described in detail in the study protocol of FtV [60].

**Table 1:** Overview of “Feel the ViBe” elements.

Element	Extra information	Restrictions
General information on exposure to family violence	Information by age (under 12 years, 12-17 years, 18-25 years, and parents) and by subject.	Public
Research information and disclaimer	Information for participants and parents about research, safety, and privacy.	Public
Information on sponsoring	Homepage, bottom left.	Public
Contact page	Option to register or ask questions to the community manager or researchers.	Public
News page	Twitter newsfeed included. The news page states important information for participants such as major bug fixes, changes in content, and scheduled maintenance.	Public
Emergency exit	A button on every page directing participants to a search engine without option to go back in the browser.	Public
Electronic consent for participants	Consent is necessary to get access to other elements behind login	Available after first login.
Electronic consent for parents	Consent is necessary for participants under 16 years old to get access to other elements behind login	Accessible by email with a code.
Questionnaires	Questionnaires will be activated in the personal menu. Questionnaires can be filled out one-by-one. Whenever possible, adaptive questioning is being used to make the burden as low as possible. There is a maximum of 15 questions per page. All items need to be filled out to submit a questionnaire. Participants cannot review their answers.	Available after first login, and every 6 weeks.
Personal menu	Menu for the participants with overview to all the available elements, access to the participants profile, digital testament, research information, and contact information.	Login needed
User profile	The profile contains information on the participant, being: full name, nickname, avatar, sex, age, contact details, and contact person. Only the nickname is available for other participants. The participant can choose a theme for the layout.	Login needed
Digital testament	The digital testament is required to fill out and lets participants choose how their data must be handled if they stop their participation.	Login needed
Ask the expert	Option to ask questions by email to several experts, including a general practitioner, a sexologist, a psychologist, and an expert in the field of family violence. Participants can also contact the community manager for general questions and questions regarding regular health care services. Response is given within 72 h.	Login needed
Forum	The forum is meant to stimulate peer support. The community manager moderates the forum and stimulates contact.	Login needed
Chat	Every 2 weeks we will offer a chat session for the participants with a specific theme and supported by an expert and the community manager. Every other week there will be an unguided chat.	Login needed
Information	Depending on the age in the profile, participants have access to tailored information about partner violence, sexual health, reproductive health, relations, and health care.	Login needed
Facts and figures	In a 12-week cycle, participants receive a 1-sentence fact of figure about family violence, sexual health, or reproductive health every day on their mobile or by email.	Consent needed

### Primary Outcomes

The Impact of Event Scale (IES) was chosen to measure PTSD symptoms. The IES is a short set of 15 questions measuring the impact of events and the amount of distress associated with events. It comprises the subscales Intrusion (8 items, mean alpha=.86) and Avoidance (7 items, mean alpha=.82) [67-70].

The Depression and Anxiety subscales of the Symptom CheckList-90-R (SCL-90-R DEP and ANX) were chosen to measure an improvement in symptoms of depression and anxiety. The SCL-90-R DEP and ANX subscales measure symptoms of depression and anxiety during the previous week on a 5-point Likert scale. Both subscales showed good convergent and divergent validity and high internal consistencies. The SCL-90-R is validated for participants aged 12 years and older. The Depression subscale comprises 16 items (alpha=.90), and the Anxiety subscale comprises 10 items (alpha=.88) [71].

### Feasibility and General Measures

The General Questionnaire (GQ) collected data on demographics and other (health) care and support received.

The Web Evaluation Questionnaire (WEQ) contained questions about content, layout, and the perceived effectiveness and usefulness of the website. The WEQ was meant to identify issues for further improvement of FtV, collect possible facilitators and barriers for implementation, and evaluate if the website met the expectations of the target group.

“Use” was measured by the collection of quantitative data (login frequency and duration, visited pages, and visitor numbers) and qualitative data (forum and chat entries and questions asked to the experts) and was monitored on a continuous base [63,72].

Qualitative data were collected from open-ended questions in questionnaires and from CM reports, including their daily activities and actions [73-75].

### Data Collection

Participants in both groups were asked to complete the IES and SCL-90 DEP and ANX at baseline (T0) and every 6 weeks until 24 weeks after inclusion (T1-T2-T3-T4). The GQ was completed at baseline and after 12 (T2) and 24 (T4) weeks. The WEQ was completed 12 weeks after receiving full access to FtV, T2 for the intervention group and T4 for the control group. According to protocol, participants received up to 2 reminders if they did not complete a questionnaire within 1 week. All outcomes were self-assessed through Web-based questionnaires. All other data were collected on a continuous basis from Google analytics, content management system (CMS) logs, and data files (Figure 1).

### Sample Size

As described in the study protocol, we calculated the sample size from studies on eHealth also using the IES as primary outcome measure [44,45,76,77]. From these studies we estimated that we needed 9 participants in each group after 12 weeks. Considering the relatively high effect sizes found in these studies and the expected loss to attrition, we aimed to include 50 participants for each group. We managed to include 93 participants within the inclusion period, of which 40 participants completed their baseline questionnaires and 17 participants (8 intervention, 9 control) completed at least their T1 and T2 questionnaires. This means that we did not reach our estimated sample size of 9 participants in each group.

### Changes to Protocol and Unexpected Events

No changes were made to the intervention. During the study period, every month between 1:00 AM and 6:00 AM there were brief (5-15 min) security updates. There were 5 bug fixes, three for the chat function, one for the login procedure, and one for the firewall. Bug fixes were planned in the night and lasted a maximum of 30 min. No content was changed. The procedure book for the intervention had minor changes 2 times during the intervention, none of which had visible impact for participants.

In the study protocol, we stated that all questionnaires were obligatory. In practice, however, due to ethical concerns, we did not exclude participants if they didn’t complete all baseline questionnaires. We were not able to report results on routine outcome measurements (ROM); participants often used the emergency exit instead of logging out, making results on ROM unusable. The adapted version of the “Seks onder je 25e” (Sex-under-25) questionnaire was hardly completed by participants because they did not feel comfortable with the subject. Therefore, we could not use the questionnaire and excluded it from analysis.

### Data Analysis

#### Effectiveness

Participant characteristics were collected from the contact forms and GQ. To maximize feelings of safety and anonymity, data on FV exposure was not actively collected. Therefore, data on FV exposure was collected from qualitative data. Characteristics of the intervention and the control group were compared to check whether randomization resulted in similar groups.

Initially, we planned an intention-to-treat (ITT) analysis, in which the intervention group, irrespective of Web-based activity and adherence to protocol, was compared with the control group at T0, T1, and T2. However, imputing missing data, which is essential to perform a full ITT analysis, is an important problem we expected to happen in an Internet-based self-support intervention for AYAs because of loss to attrition. After consultation of a medical statistician, we decided not to use imputation techniques for missing data, as this would lead to a too large level of uncertainty in the relatively small group.

Therefore we primarily performed a complete case analysis using univariate analysis of variance (UNIANOVA) and mixed model analysis in statistical package for the social sciences (SPSS version 22; IBM Corp) to analyze any effects of using FtV in the first 12 weeks, based on the randomization in the initial group (ITT).

Second, we performed a pre-post *t* test analysis for all participants being an active user during 12 weeks, independent of their original randomization group. *P* values <.05 were considered statistically significant. Whenever relevant, age-related differences were included.

#### Feasibility

Feasibility studies aim to study several areas of focus to be able to fully understand the strengths and weaknesses of a new intervention: the potential for success when implementing it in the real-world. Feasibility was analyzed using several areas of focus, as suggested by Bowen et al (2009) [78,79]. Participant characteristics and Google analytics were used to assess expressed interest. Quantitative usage data were used to assess demand. First we focused on intention to use, followed by an analysis of actual use to assess continued use. Both individual elements as total usage time were included. Quantitative data were supported by self-reported qualitative data on use.

Quantitative and qualitative satisfaction assessed acceptability. Appropriateness was evaluated comparing user’s wishes and needs, including safety, with expected goals as reported in the GQ. CM reports and a costs analysis were used to investigate possibilities for implementation in an uncontrolled design.

All qualitative data were analyzed using a thematic coding approach. Two researchers (KRN and SLFW) read and coded all qualitative log files. Consensus was reached in mutual discussion. Coded fragments were grouped in themes using feasibility measures as overall guideline. Any disagreements in coding were discussed in the supervising research team. Quotes are given a quote number corresponding with the number in Table 6.

**Table 2:** Participant characteristics as measured in the General Questionnaire (n=40).

Participant characteristics		Intervention (N=20)	Control (N=20)	*P* value
**Age (years)**					
	Mean age	18.40 (SD 3.62)	18.20 (SD 3.02)	.85
	12-17	8	10	.54
	18-25	12	10
**Sex**				.32
	Male	1	0	
	Female	19	20	
**Country of birth**				.04
	Netherlands	20	16
	Belgium	0	4
**Country of birth** **mother**				.24
	Netherlands	17	12
	Belgium	0	5
	Other	3	3
**Country of birth** **father**				.55
	Netherlands	17	13
	Belgium	0	3
	Other	3	4
**Religion**				.29
	Christianity	11	9
	Islam	1	1
	No religion	5	8
	Other	3	2
**Importance of religion**				.06
	Not important	11	16
	A bit important	7	4
	Very important	2	0
**Employment**				.54
	Full time education	10	11	
	Employed	3	3	
	Both studying and job	6	5	
	Disabled	1	1	
**Education**				.65
	Lower education	3	3	
	Middle education	9	5	
	Higher education	8	12	
**Current relationship**				.38
	Boyfriend	5	7	
	Girlfriend	0	1	
	Dating	1	1	
	None	14	11	
**Living situation**				.71
	At home with parents	14	15
	With partner	1	0
	Alone	3	4
	Sheltered housing	2	1
**Alcohol use**				.90
	Daily	1	0
	>1 time/week	3	5
	<1 time/week	6	6
	Never	10	9
**Smoking**				.67
	Yes	1	2
	Before	3	1
	No	16	17
**Use of drugs**				.35
	>1 time/week	1	0
	<1 time/week	1	1
	Never	18	19

### Ethics

This study was conducted in the Netherlands and was registered in The Netherlands National Trial Register (NTR3692).

The Committee on Research Involving Human Subjects of the Radboud University Nijmegen Medical Centre (Dutch initials: CMO) has assessed this study and judged that the study does not fall within the remit of the Medical Research Involving Human Subjects Act (WMO). Therefore, the study could be carried out (in the Netherlands) without approval by an accredited research ethics committee (2011/053. NL nr 35813.091.11. March, 16th, 2012).

## Results

### Effectiveness

100 participants, of which 9 male and 91 female participants with a mean age of 18.55 (SD 4.23) were included. After randomization, 7 participants were excluded. Fifteen participants in the intervention group and 21 participants in the control group did not give electronic consent, meaning that 31 out of 46 participants in the intervention group and 26 out of 47 participants in the control group started to use FtV. Of these participants, 20 participants in each group completed their baseline questionnaires and were included in the analysis (Figure 1). All these participants were exposed to FV. Eight participants in the intervention group, compared with 7 in the control group, were not only exposed but also a direct victim of FV. Overall participant characteristics are in Table 2.

Of the 40 participants who completed their baseline participants, 17 participants completed all questionnaires. There were no significant differences in age, sex, and type of violence between the participants who completed T0 (n=40, mean age 18.38 [SD 3.23]) and the participants who did not (n=17, mean age 16.94 [SD 3.49]) (*P*=.14). There were no significant differences in patient characteristics between the intervention and the control group at T0 (Table 2), except for country of birth and baseline measurements: The intervention group scored significantly lower scores on the IES with a mean score of 33.95, interpreting as “powerful impact event,” whereas the control group scored 45.60, interpreting as “severe impact event” (*P*=.01). Dropouts, participants who completed T0 but not T2 (n=15), were compared with completers (n=25); there were no significant differences at T0 for the intervention group, but in the control group, dropouts scored higher on the IES avoidance subscale (not significant, ns) and significant lower on the IES intrusion subscale (16.67 [SD 9.69] vs 24.64 [SD 5.40], *P*=.03).

We performed a UNIANOVA to correct for the differences on baseline scores. This showed no overall significant differences between T0 and T2 (Table 3).

**Figure 2:**
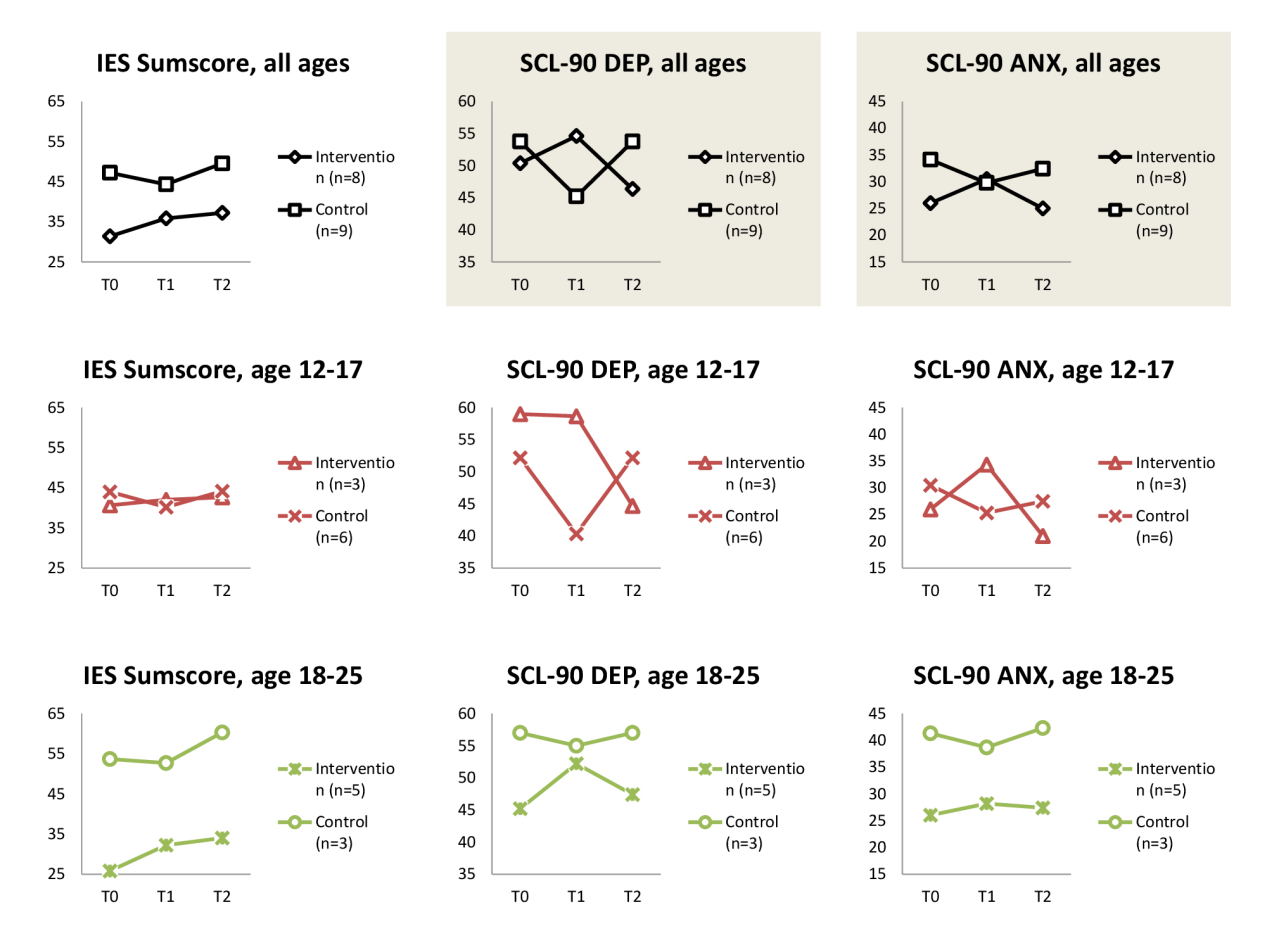
Course in time for intervention compared with control group participants, gray area meaning that course difference is significant (*P*<.05).

A mixed model analysis showed that the course of intervention participants is significant different for control participants on the SCL-DEP and ANX subscores. Repeating the analysis for separate age groups, results follow the same course, although results are no longer significant (Figure 2).

According to the protocol, the control group received full access to FtV after 12 weeks (Figure 1). Considering their T2-T4 measurements as T0-T2, we performed pre-post test measurements to further investigate the aforementioned findings. Considering them as two separate intervention groups, we saw that both groups improve on the SCL-90 DEP and ANX subscales between T0 and T2. For group 2, who had been on a waiting list for 12 weeks before receiving access, these differences were significant for all measurements (Table 4).

#### Qualitative Effectiveness

Of the participants who completed the WEQ, 58% (11/19) answered to an open-ended question that they were doing okay-to-fine at that moment. To investigate subjective efficacy, we asked participants whether FtV was helpful for them: on a 1-5 Likert scale, the helpfulness score was 3.16, with 42% (8/19) of participants saying that FtV was helpful or very helpful and 26% (5/19) saying that FtV was partly helpful. Additionally, 5% (1/19) said that FtV was not helpful at all. Being asked to motivate, participants said that “meeting others who experience violence” (58%, 11/19), “recognizing other’s stories” (58%, 11/19), and “being able to ask questions about violence” (58%, 11/19) was helpful. “Talking about their personal situation” (53%, 10/19) and “finding out if something was normal or not” (53% 10/19) were also indicated as helpful. Forty-two percent (8/19) of all participants said that giving support to others was also helpful for themselves.

### Feasibility

#### Demand and Use

Google analytics data showed that 18,534 visitors visited the public website of FtV from June 1, 2012 to January 1, 2014. About 65.00% (12,047/18,534) of the users visited FtV only once with a bounce rate (percentage of users that leaves feel-the-vibe.nl after the first page they visit) of 58.00% (6987/12,047), which is considered good. The remaining 35.00% (6487/18,534) were recurrent visitors, visiting an average of 7.5 pages in 7 min per visit in the public part of the website. Most visitors accessed FtV either directly (32.00%, 5930/18,534) or by Google (47.00%, 8711/18534). A minority accessed FtV trough links on websites giving general information on child abuse or FV. Furthermore, 84% (16/19) of all participants who completed the WEQ agreed that FtV was easy to find on Google. A total of 194 visitors submitted a contact form. From these, 100 concerned eligible participants. Seven participants were excluded after randomization. Additionally, 31 out of 46 participants in the intervention group and 26 out of 47 participants in the control group started to use FtV (Figure 1). Participants under 16 years (needing parental consent), did not give less consent compared with older participants (65% [13/20] compared to 62% [44/71], respectively). Young adults (18-25 years) tend to give less often consent (55%, 27/49) than adolescents (12-17) (71%, 30/42), however, not significant (*P*=.13). After giving consent, 29 participants (among which all male participants) did not use the intervention. Four users used the intervention for 12-24 weeks. The remaining 24 users used FtV for 24 weeks or longer. There were no significant differences between adolescents (12-17) and young adults (18-25) in login count or session time, but adolescents (12-17) used the chat significant more than young adults in their first 12 weeks of access. Overall participant activity can be found in Table 5. Table 5 shows usage data of participants during their first and second 12 weeks on the Web.

**Table 3:** Effect of “Feel the ViBe” after 6 weeks (T1) and 12 weeks (T2) controlling for T0 (intention-to-treat, complete case). UNIANOVA calculating mean score, controlling for T0 to correct for differences between groups.

Outcome	Time	Intervention (n=8)	Control (n=9)	B	*P*
		Mean (95% CI)	Mean (95% CI)		
Impact of Event Scale					
	T1^a^	42.34 (34.71-49.97)	38.59 (31.44-45.73)	3.75	.48
	T2^a^	42.89 (34.38-51.40)	44.54 (36.57-52.51)	−1.65	.78
Symptom CheckList-90-R, Depression subscale					
	T1^b^	56.44 (48.60-64.29)	43.61 (36.21-51.00)	12.84	.02
	T2^b^	47.70 (38.73-56.67)	52.60 (44.15-61.05)	−4.90	.41
Symptom CheckList-90-R, Anxiety subscale					
	T1^c^	34.67 (28.16-41.18) 26.07 (19.96-32.18)	8.60	.07	
	T2^c^	29.18 (25.64-32.71)	28.73 (25.41-32.05)	.45	.85

^a^Mean score at T1 and T2 corrected for the overall mean score at T0 = 39.76 (n=17).

^b^Mean score at T1 and T2 corrected for the overall mean score at T0 = 52.18 (n=17).

^c^Mean score at T1 and T2 corrected for the overall mean score at T0 = 30.29 (n=17).

**Table 4:** Pre-post test analysis (n=14).

Outcome	Group 1 Mean (SD) (n=8)^a^			Group 2 Mean (SD) (n=6)^b^			All Mean (SD) (n=14)		
	T0	T2	*P*	T0	T2	*P*	T0	T2	*P*
IES^c^ sum	31.38 (18.25)	37.25 (15.12)	.07	52.83 (9.77)	36.33 (10.78)	.02	40.57 (18.37)	36.86 (12.94)	.36
SCL-90 DEP^d^	50.38 (17.70)	46.38 (15.93)	.47	57.17 (11.99)	42.50 (10.71)	.01	53.29 (15.36)	44.71 (13.59)	.03
SCL-90 ANX^e^	26.00 (9.18)	25.00 (8.91)	.54	34.00 (9.82)	27.67 (8.34)	.045	29.43 (9.97)	26.14 (8.45)	.046

^a^Originally randomized to intervention group.

^b^Originally randomized to control group, considering T2-T4 measurements (after receiving full access) as T0-T2.

^c^IES: Impact of Event Scale.

^d^SCL-90 DEP: Symptom Checklist-90 Depression subscale.

^e^SCL-90 AUX: Symptom Checklist-90 Anxiety subscale.

Actual use measured on the Web corresponded well with self-reported data by participants. The 19 participants who completed the WEQ reported a mean Web-based time of 2.83 (2-3 times a week), with a mean Web-based session time of 36 min. Participants said they visited the forum and chat most and information on sex and relations least often, which corresponds with the quantitative usage data.

**Table 5:** Participant activity for all active users ≥24 weeks (n=24). It shows usage data of participants during their first and second 12 weeks on the Web.

Activity		0-12 weeks access	12-24 weeks access	*P* value
		Mean (SD)	Mean (SD)	
Total sessions (n)					
	All ages (n=24) 50 (45,57)	16 (15.63)	<.001	
	Age 12-17 years (n=11)	43 (45.56)	19 (15.29)	.05
	Age 18-25 years (n=13)	55 (46.72)	13 (15.89)	.001
	*P*	.54	.32	
Session duration minutes					
	All ages (n=24)	27 (14.12)	23 (14.37)	.35
	Age 12-17 years (n=11)	31 (13.46)	24 (14.14)	.32
	Age 18-25 years (n=13)	24 (14.36)	21 (15.00)	.71
	*P*	.23	.59	
Chat per session					
	All ages (n=24)	0.54 (0.28)	0.61 (0.34)	.16
	Age 12-17 years (n=11)	0.73 (0.21)	0.69 (0.26)	.31
	Age 18-25 years (n=13)	0.37 (0.22)	0.55 (0.40)	.04
	*P*	<.001	.34	
Forum per session					
	All ages (n=24)	0.69 (0.32)	0.65 (0.38)	.56
	Age 12-17 years (n=11)	0.59 (0.35)	0.49 (0.45)	.09
	Age 18-25 years (n=13)	0.78 (0.27)	0.79 (0.24)	.86
	*P*	.15	.06	
Chat and/or Forum per session					
	All ages (n=24)	0.91 (0.12)	0.91 (0.15)	.99
	Age 12-17 years (n=11)	0.95 (0.06)	0.91 (0.09)	.18
	Age 18-25 years (n=13)	0.89 (0.15)	0.91 (0.19)	.66
	*P*	.21	.98	
Information per session					
	All ages (n=24)	0.10 (0.09)	0.05 (0.07)	.02
	Age 12-17 years (n=11)	0.11 (0.11)	0.05 (0.07)	.09
	Age 18-25 years (n=13)	0.09 (0.07)	0.05 (0.08)	.12
	*P*	.64	.78	
Ask-the-Expert per session					
	All ages (n=24)	0.05 (0.05)	0.05 (0.11)	.91
	Age 12-17 years (n=11)	0.06 (0.06)	0.04 (0.07)	.27
	Age 18-25 years (n=13)	0.04 (0.04)	0.06 (0.14)	.60
	*P*	.33	.65	

#### Acceptability

Results from the baseline GQ and WEQ show that first impressions of FtV were positive—participants were enthusiastic (47%, 9/19) or felt that FtV was made especially for people in their own situation (32%, 6/19). At T0, “contact with fellow sufferers” was said to be both the most important wish and the most important need for their participation in FtV, followed by “someone listening without taking action right away.” “Gathering information” and “receiving support or advice” were other frequently named wishes, also expressed as need. “Receiving help from Web-based health care providers” was indicated third most expressed wish for FtV but was not named as a need. Asked to goals, both in open and multiple-choice questions, the same 5 categories were named as most important (Table 6, Q1).

**Table 6:** Qualitative quotes by participants and community managers.

Question number	Participant number	Age	Source	Quote
Q1	290	19	GQ^a^	Giving people information and helping or supporting. And chatting with people who have gone through the same en by doing this helping each other.
Q2	209	20	WEQ^b^	Keep up the good work. I wish there were more people like you guys.
Q3	204	24	WEQ	I think the website is super, good initiative. You can find good and clear information. For me though I am not feeling a real connection or click with the others, which I think is because of the age difference. I pity that.
Q4	241	17	WEQ	The professional and the other participants answer your questions directly and help you immediately, and it feels like a relieve when you had a conversation like that.
Q5	207	21	WEQ	I don’t think this is relevant, sex has, to my opinion, not always something to do with domestic violence. Sometimes it seems that, if it concerns adolescents, there always has to be a part about sexual education
Q6	228	17	WEQ	I feel safe because there is an emergency exit and your contact details are being stored, so when you really need help they can help you and they answer your questions personally.
Q7	209	20	WEQ	The Community Manager is very committed and gives you a warm feeling. I am not scared at all that she will tell anybody or forces me to do anything (which I know from my own experience).
Q8	CM^c^1	51	CM report	A strength of FtV is the time for participants to build a trusting relation and I fear this is not possible with a student.
Q9	CM3	26	CM report	I feel that FtV works, because it is not seen as healthcare by the participants, being not linked to any kind of organisation (...) thus feeling safe.

^a^GQ: general questionnaire.

^b^WEQ: Web evaluation questionnaire.

^c^CM: community manager.

Whereas support and information were very important, direct action was less asked for: Only one in five participants wished the violence at home would stop because of their participation and no participants identified it as a need. “Stopping the violence” (21%, 4/19) and “getting someone to receive help from a health care provider” (16%, 3/19) were chosen least often as goals of FtV.

After being on the Web for 12 weeks, participants expressed mostly joy about the existence of FtV (58%, 11/19) but only 16% (3/19) felt FtV had already helped them solve their problems. FtV was rated a mean score of 7.47 (range 6-9) on a 1-10 Likert scale (Q2, Q3). Overall, content, language, structure, user interface, and layout were rated good. Guided chat (42%, 8/19) and forum (37%, 7/19) were chosen the best parts of FtV because of the possibility to share stories and ask questions (Q4). All theme chats were valued positively, including the professional contact options and information about FV. Least valued were the unguided chat and informational pages, especially on sex and relations (Q5).

#### Safety

Safety was named second most frequently as need for FtV, direct after “contact with fellow sufferers.” All participants said they felt safe, because of the relative anonymity (26%, 5/19); the emergency exit (21%, 4/19); acknowledgement and recognition by peers and professionals (21%, 4/19); the enjoyable, pleasant, and cozy environment (16%, 3/19); the approach and availability of the CMs (11%, 2/19); reliability of FtV (5%, 1/19); the technical security protocols (5%, 1/19); and/or having a safety protocol with contact details in case of participant danger (5%, 1/19) (Q6, Q7).

Seventy-four percent (14/19) of the participants thought that a personal message with the results of their questionnaires would make them feel even safer and 16% (3/19) thought that a better explanation of the safety protocols or 1 phone call with the CM could improve safety. In general, however, participants would not want more of their details to be known. Parent consent was perceived negatively.

#### Implementation and Practicality

CMs spent an average of 14 h a week on FtV. A guided chat lasted a mean 100 min, an individual chat, held only in case of danger or request for immediate help, 70 min. Around 70% (136/194) of all contact forms were answered within 36 h, the remaining 30% (58/194) within the maximum 72 h. All emergency messages were answered within 24 h, mainly within 6 h. CMs were asked to name essentials for success (Table 7). If these cannot be met, risks indicated concerned mainly harm for the participant and suboptimal care, but also a risk of burn-out for the CM, when time investment and commitment are too high.

**Table 7:** Essentials for successful implementation of “Feel the ViBe.”

General essentials^a^	Community manager characteristics^b^
Unrestricted access to the Internet for community managers	Computer and Internet skills
Unrestricted access to the internet for participants	Trained in giving Web-based support and help
Safety protocols to handle the variety of problems and participants	Trained in assessing safety during Web-based communication
24/7 availability in case of emergency (ICE) from pool of community managers	Flexible and able to adapt quickly in time, nature, and language of help provided
Colleagues to discuss participants’ situation	-

^a^Community managers were asked to name elements essential for FtV.

^b^Community managers were asked to identify personal characteristics of community managers essential for FtV.

An estimation of costs of development, execution, and future implementation was made calculating the costs from 2012 to 2014 (Table 8). Costs were calculated using actual costs for 2012, 2013, and 2014 (mean score is given when applicable).

**Table 8:** Estimation of costs for implementation of “Feel the ViBe.” Costs were calculated using actual costs for 2012, 2013, and 2014 (mean score is given when applicable).

Category	Necessity	Costs (US $)	Recurrent?
Intervention	Development only^a^	50,000	One-time only
Mobile app	Optional	10,000	One-time only
Hosting, security, and updates	Essential	12,000	Yearly
Internet, mobile and office resources, for example, computer, mobile phone, subscriptions.	Essential^b^	2000	Yearly
Salary costs: community manager (20h/week), coordinator (8h/week)	Essential^c,d^	48,000	Yearly
Professionals on consultation base	Essential	6000	Yearly

^a^Adaptation will cost about 10-25% of development costs, depending on need for translation.

^b^Costs based on minimally needed resources.

^c^Costs are calculated for Dutch salary norms, meaning that the actual costs can vary across countries depending on the salary norms.

^d^Costs could be lowered using trained volunteers or medical students.

CMs agreed that their work might be done by a student or volunteer with a background in health care, although they feared that the continuity of care would be endangered (Q8). Above that, they felt that the strain on personal life and the risk to become too personally involved were rather high.

To identify the position of FtV within the current field of health care, we asked participants in the baseline GQ to identify all possible sources of help when encountering FV. The mentor (73%, 29/40) and the school counselor (60%, 24/40) were the most named persons. The family physician was the most well-known health care provider—55% (22/40) knew how to get help there. One third of the participants did not know they could ask for help at the national emergency line and the police. At baseline, 18 participants received another form of health care, being mostly informal care from a mentor (15%, 6/40) or school counselor (13%, 5/40), or formal care from a psychologist (10%, 4/40). After the first 12 weeks of the intervention, 24 participants filled out the follow-up GQ. Two-thirds of them (15/24) started regular health care: mental health care (10/15), general practice (1/15), counselor or mentor (4/15), youth care (2/15), other Web-based help (2/15).

CMs agreed that FtV should be integrated within public health care or as part of the national services for FV and child abuse instead of existing health care institutions: they feared limited availability and accessibility, lack of anonymity, legal rules and/or regulations, and insurance requirements. CMs thought this would lower feelings of safety and enlarge the threshold for participation (Q9).

## Discussion

### Principal Findings

AYAs exposed to FV need health care in an early phase to deal with the consequences of this exposure. FtV was developed as a low-threshold, Internet-based, self-support intervention to provide AYAs exposed to FV with (peer) support and information, to lower the threshold to regular health care for those who need this. This study investigated first, the efficacy of FtV and second, the feasibility of FtV using a mixed methods approach. To our knowledge, we are the first to use this approach for the evaluation of an eHealth method in the field of FV. No strict conclusions on efficacy could be drawn as the participant rate was rather low. We conclude that the acceptability for FtV, including satisfaction and safety, was good. In the following paragraphs we would like to highlight some of the most important findings.

#### Effectiveness

Overall we feel that FtV is a suitable and satisfying intervention for the target group. However, in a small population with large differences in characteristics, it is difficult to find meaningful effects. Besides, we did not reach the sample size (17 instead of 18 participants). In our study sample, mean scores at baseline were very high, indicating a potential post-traumatic stress disorder in almost all participants. Intervention participants worsened in their scores before improving to levels above their start level. Control group participants showed the other way around. Further research exploring possible explanations is needed: One could hypothesize that control group participants improve because they are happy to have sought help, stepping up to a higher level of change, whereas intervention participants become aware of their situation and the abnormality of it. Depressive thoughts could increase as well as anxiety out of fear for the consequences of seeking help for their situation.

Overall, it is well known from literature that AYAs exposed to FV suffer from mental health problems, comparable with being a victim of child abuse themselves [6,8,25,80-82]. Treating this may take much longer than 6 or 12 weeks. Considering that, results indicating that SCL-90 ANX and DEP scores improve significantly within the second 6 weeks of access are promising. The subjective effect is high: participants feel helped by their participation and two-thirds of all participants started other health care while being a participant of FtV. Therefore, we think that FtV is a promising intervention, although future research should study prolonged effects over time.

#### Stages of Change

Characteristics of participants registering for FtV are diverse. All participants were exposed to FV. Fifteen participants were not only exposed but also a victim themselves, which is in accordance with literature [4]. Wishes, needs, and goals, however, are quite uniform and mainly directed at support, information, and safety, which is in line with other studies [28,30,31,33,83-86].

As we described in the introduction, we used the transtheoretical model of behavior change of Prochaska and Di Clemente [24] to categorize potential participants of FtV and hypothesized that most of them would be in a precontemplation phase, whereas regular health care is mostly directed at participants in an action phase. Results show us that the bounce rate is relatively low, meaning that many visitors visiting FtV want to know more about the subject. However, only a small part of the visitors to the public website of FtV send a contact form. This corresponds with a precontemplation phase; recognizing that their situation at home is different, participants in this phase might be looking for information only. This could be a too early phase to participate in FtV. Therefore, it is important to optimize access to FtV by extending both the public part of the intervention and Google optimization. Participants who send a contact form, use their login, and give consent might be one step further than what we hypothesized: in the preparation or planning phase. Participants in this phase are thinking about how to change their situation and are ready to take action in the near future. For them, is it important to lower the threshold to start their participation as far as possible; questionnaires and other obligated parts could hinder participants in this still early phase of change. Of the 57 participants who gave consent, only 40 participants completed their baseline questionnaires and 28 participants became active users. Therefore, interventions that mainly support participants in (precontemplation and preparation phases should focus first on safety and second on support and advice. A major pitfall in these phases is intervention requirements which enlarge the threshold for continued participation. In our target group for example, the safety protocol, including the contact person, could have enlarged the threshold for participation.

#### Attrition

eHealth interventions have to cope with the law of attrition: high levels of nonusage and dropout, which can be as high as 80-90% [61]. Considering these percentages, drop out for FtV is relatively low, with one-third of the participants still active after 36 weeks. Attrition can partly be explained by the stages of change model, but it is also important to identify possible other factors influencing nonusage and dropout since this influences the feasibility of the intervention. The threshold for participation in FtV was made as low as possible without compromising safety. However, a low threshold to participation and easy enrolment could lead to high dropout, as users fully realize the consequences of their participation only after they start participating. More strict information, personal contact (telephone, face-to-face), and making participants pay for their participation, all decrease nonusage and dropout attrition but also increase the threshold, which does not fit the early stage of change of most participants.

Another important factor in this study could be expectation management. Only after randomization and consent participants could see the intervention content and layout. Looking at the wishes and needs indicated by the participants, encountering others is very important. Finding out that there are not many participants in your first visit could lead to nonusage and dropout, meaning that new participants encounter the same problem again. We tried to solve this problem by giving the CMs more time to be on the Web and participate in discussions and chats in the first months of FtV.

Personal contact lowers dropout attrition rate. As FtV focuses on (peer) support from CMs and participants, this could have lowered the dropout attrition rate.

#### eHealth and Violence

Especially in this target group, that values safety besides peer support as most important need for participation, the advantages of an eHealth intervention are numerous. Due to its nature, it is easier to conserve anonymity and safety, lowering the threshold for participation. It is flexible and accessible. The participants lived in a large geographical range, and more often, they had difficulties visiting other health care institutions because they had to use public transport and needed money to do that. Being able to include participants from a large geographical range means that it is easier to collect enough people to give adequate support and to be economically profitable. Besides, eHealth means not having to explain absence due to therapy or other treatment requirements. To our opinion, this makes eHealth an ideal method to start health care for AYAs exposed to FV.

### Limitations

eHealth studies have to deal with a wide range of challenges and traditional designs may be less suitable for an Internet-based self-support intervention [87]. Therefore, we chose not only a traditional RCT design but also an innovative feasibility design using mixed methods. However, there are limitations that should be considered when interpreting the findings of this study. First, it is only possible to apply the results to female participants, as no male participants completed their participation. Only 9 males applied; none of which completed their questionnaires. The set-up of FtV, explicitly stating that FtV gives (peer) support and information, could be more attractive to women. Males may be less hesitating to find regular health care, having less fear to harm family and surroundings. Besides, they are also more at risk for externalizing behavioral problems, which might need a different approach.

Second, in our study the control group and the intervention group differed on their baseline measurements. There could be several explanations for this finding. Due to the nature of this study, participants knew their group when starting to fill out the questionnaires. Participants in the control group may have felt let down or could have wanted to show their need for participation. It could also mean that less severe affected participants don’t start the waiting list because their problems are not severe enough. The groups did not differ on depression and anxiety at T0.

A limitation is the fact that, due to safety protocols and according to protocol, control group participants received mEUC, meaning that they could send an emergency message when they felt they needed this. Although in none of the cases there was actual danger and all messages were handled according to protocol, we still feel that participants may experience these short contacts as support, thus influencing results.

### Implications and Recommendations

FtV is a promising intervention. Future research should focus on larger samples and investigate the optimal intervention duration.

We found that the role of the CM is very important. The CM supports, informs, and motivates, thus functioning as a coach around the clock. However, in the design of FtV, the CM was only intended to monitor, support, and inform from the background. Therefore, further research should investigate the position of the CM in a qualitative manner.

As we concluded that eHealth seems an adequate method to provide AYAs exposed to FV with care, we recommend further research to study eHealth in other groups coping with violence. One could expect results to be similar, although Internet literacy and access could be limiting potential effects.

FtV can be easily implemented without extensive resources. Nevertheless, implementing FtV within an existing health care organization, could lead to an enlargement of the threshold, or a situation in which participants who need more care are not always referred to the best health care option because of incomplete knowledge or strategy of the implementing organization. Therefore, we feel that FtV, when implemented, should be in the field of public health care or national governmental care, to provide the lowest possible threshold and long-term sustainability. FtV functions, to our opinion, best as a first step for AYAs in an early stage of change to get them ready for action and to fill the gap between exposure to FV and starting regular health care to stop violence and treat the consequences.

### Conclusions

Based on the available data, we conclude that preset goals for FtV, that is, peer support, giving information, and support in finding regular health care, have been met, making FtV a promising intervention. Reaching back to the stages of change model, we feel that participants who are in a preparation stage benefit best from FtV in gathering information and receiving support, maximizing safety, and minimizing external control. FtV may help them to move on to the action stage, get ready to start regular health care treatment, or discover that they do not need more help as FtV provides them with sufficient support.

## Appendix

Multimedia Appendix 1 Demonstration Powerpoint slides "Feel the ViBe.".

Multimedia Appendix 2 CONSORT EHealth Checklist.

## Abbreviations

AYAs: adolescents and young adults
CM: community manager
FtV: Feel the ViBe
FV: family violence
GQ: General Questionnaire
IES: Impact of Event Scale
mEUC: minimally enhanced usual care
SCL-90 DEP: Symptom CheckList-90R, Depression subscale
SCL-90 ANX: Symptom CheckList-90R, Anxiety subscale
UC: usual care
WEQ: Web Evaluation Questionnaire
