# Supplementary figures and images for: Young People, Adult Worries: Randomized Controlled Trial and Feasibility Study of the Internet-Based Self-Support Method “Feel the ViBe” for Adolescents and Young Adults Exposed to Family Violence
Source: J Med Internet Res. 2017 Jun 12;19(6):e204. doi: 10.2196/jmir.6004 (PMC5484793; doi:10.2196/jmir.6004)

## Slide 1
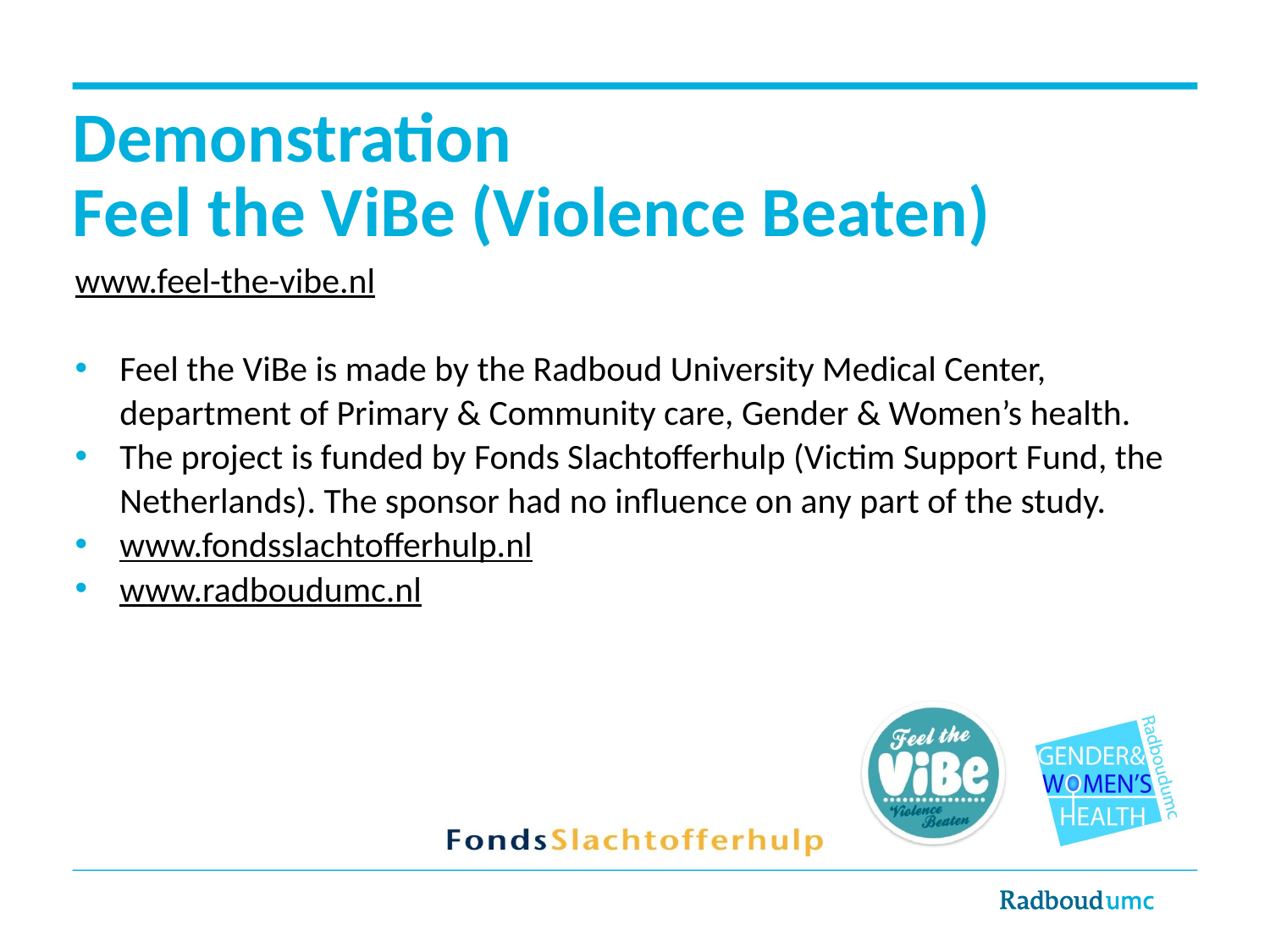

## Slide 2
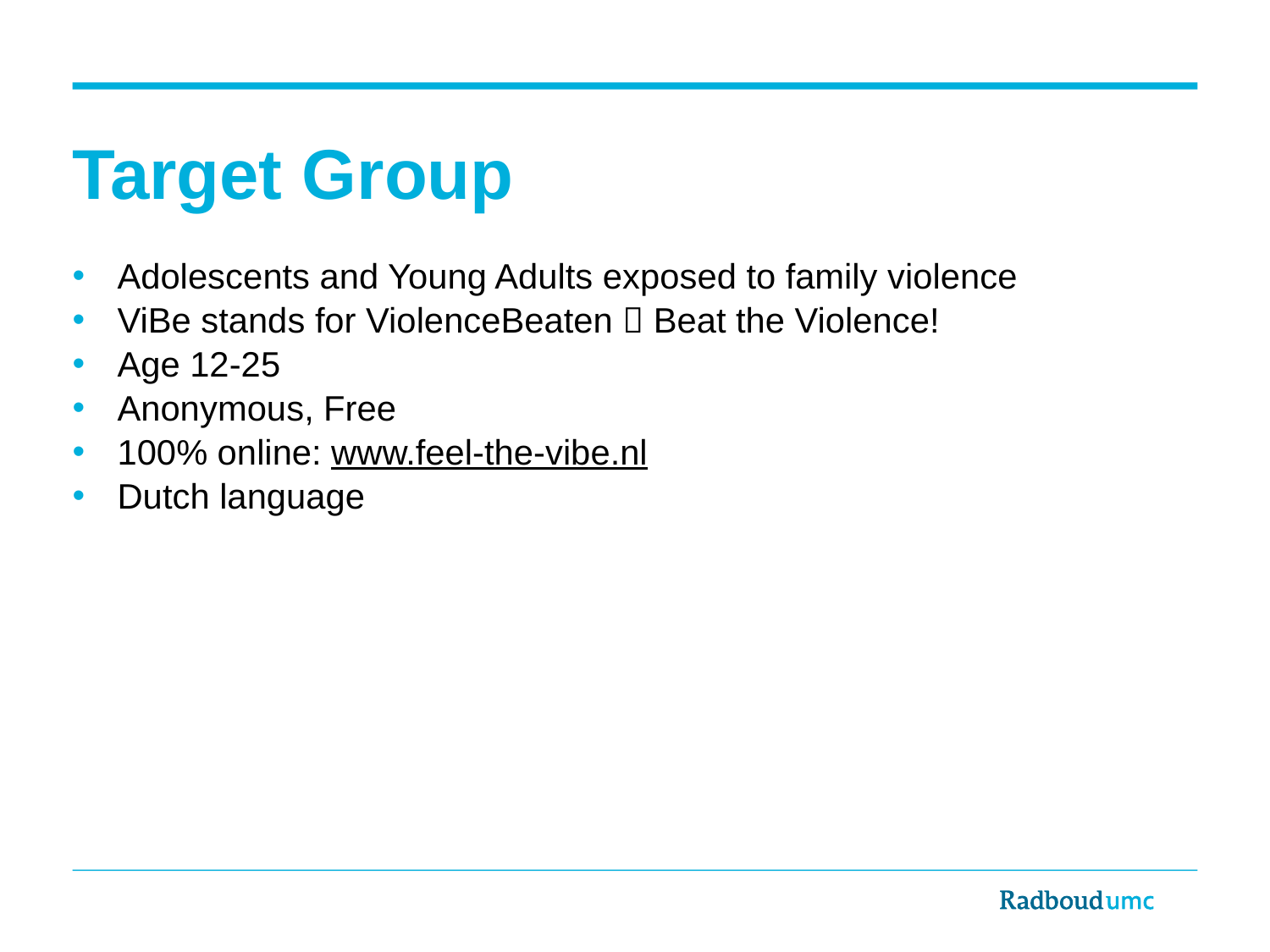

## Slide 3
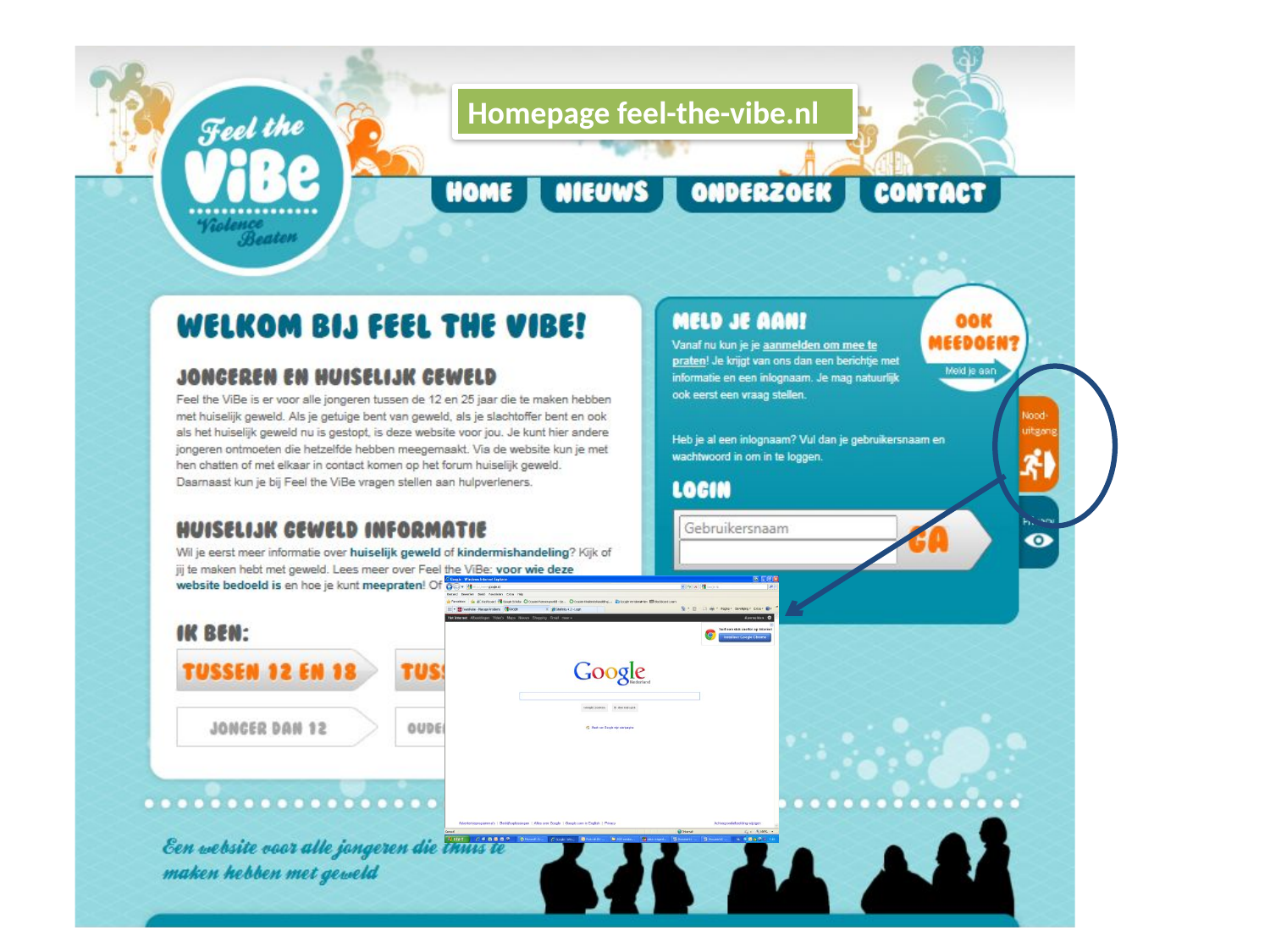

## Slide 4
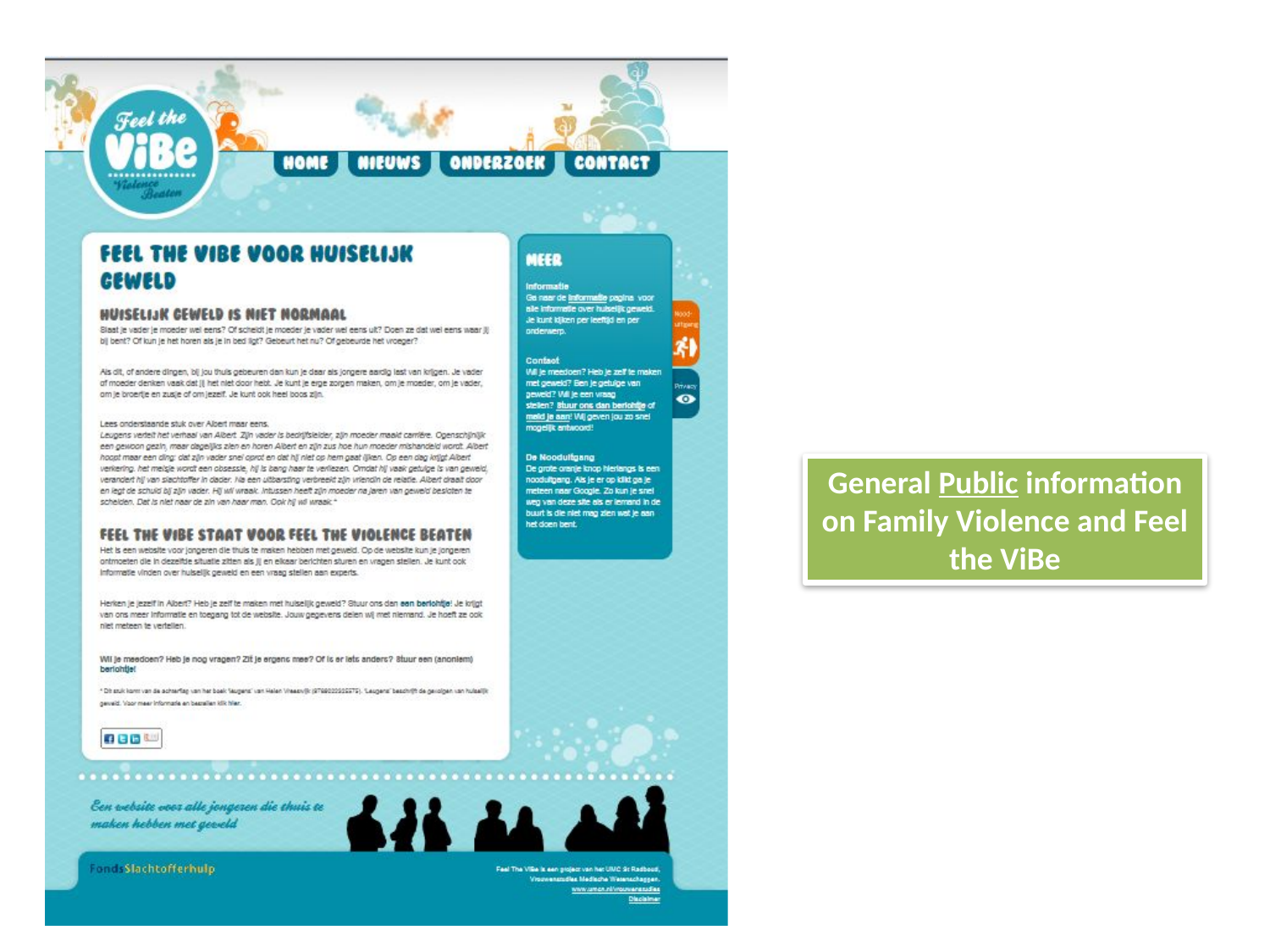

## Slide 5
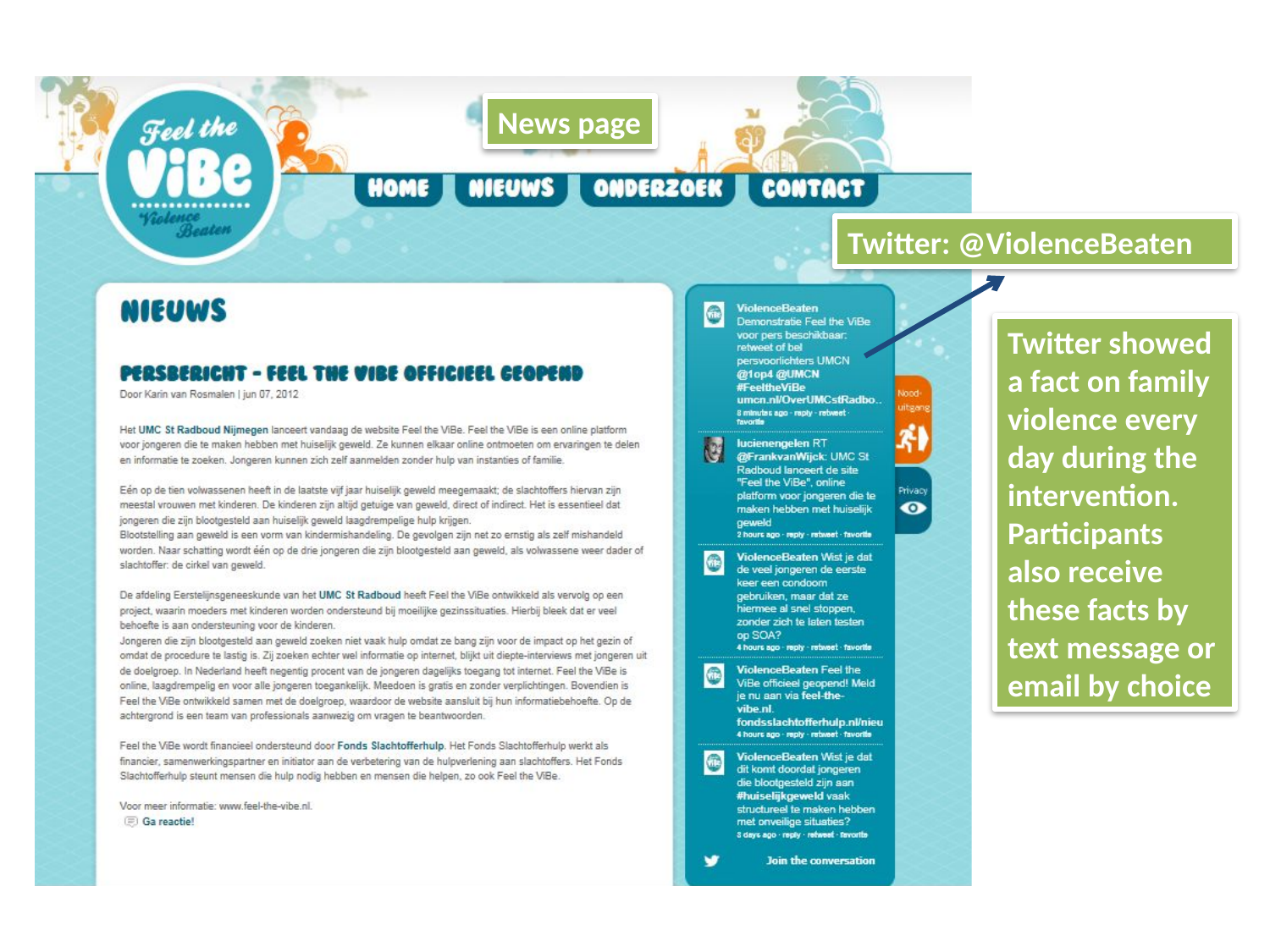

## Slide 6
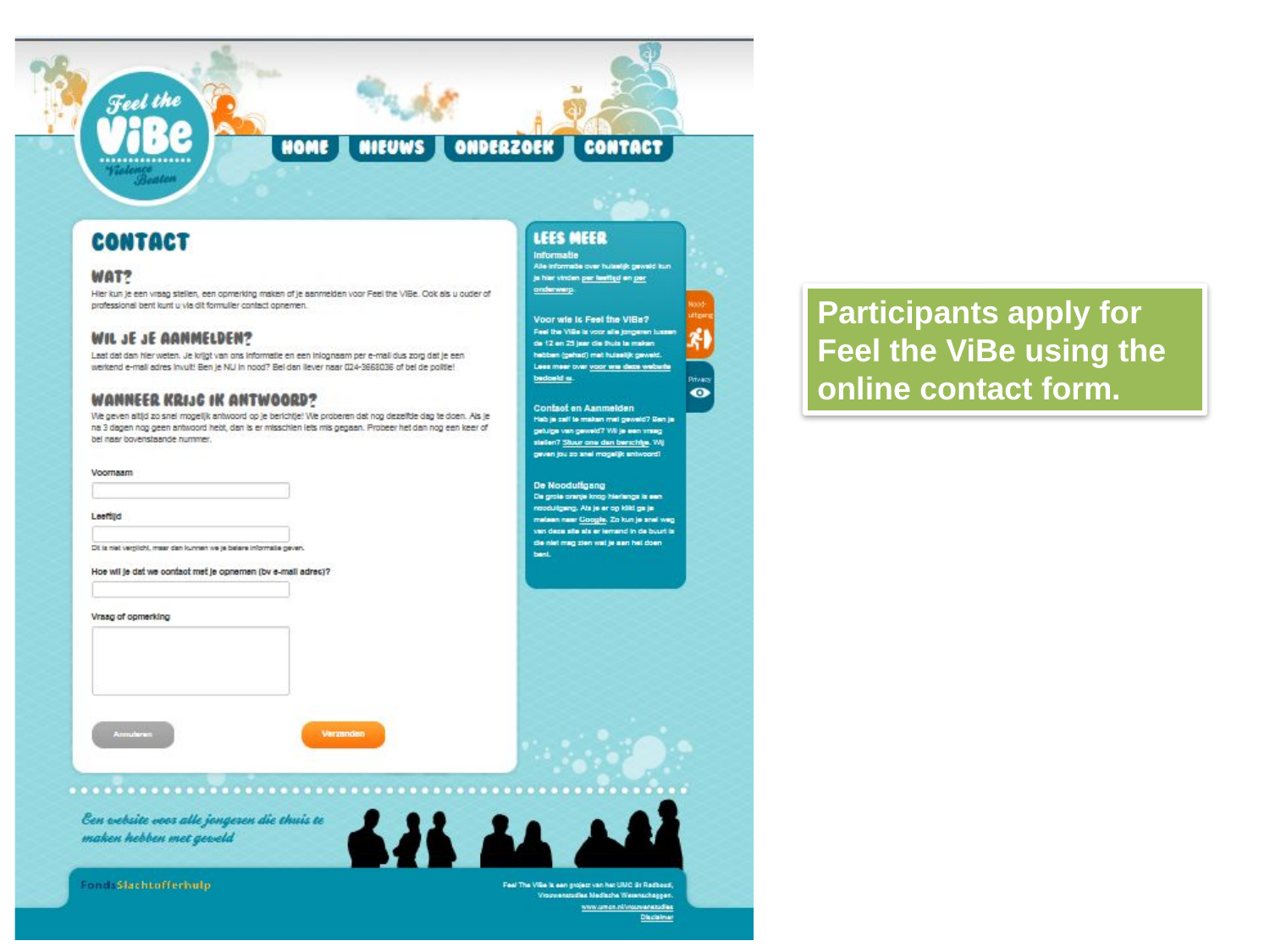

## Slide 7
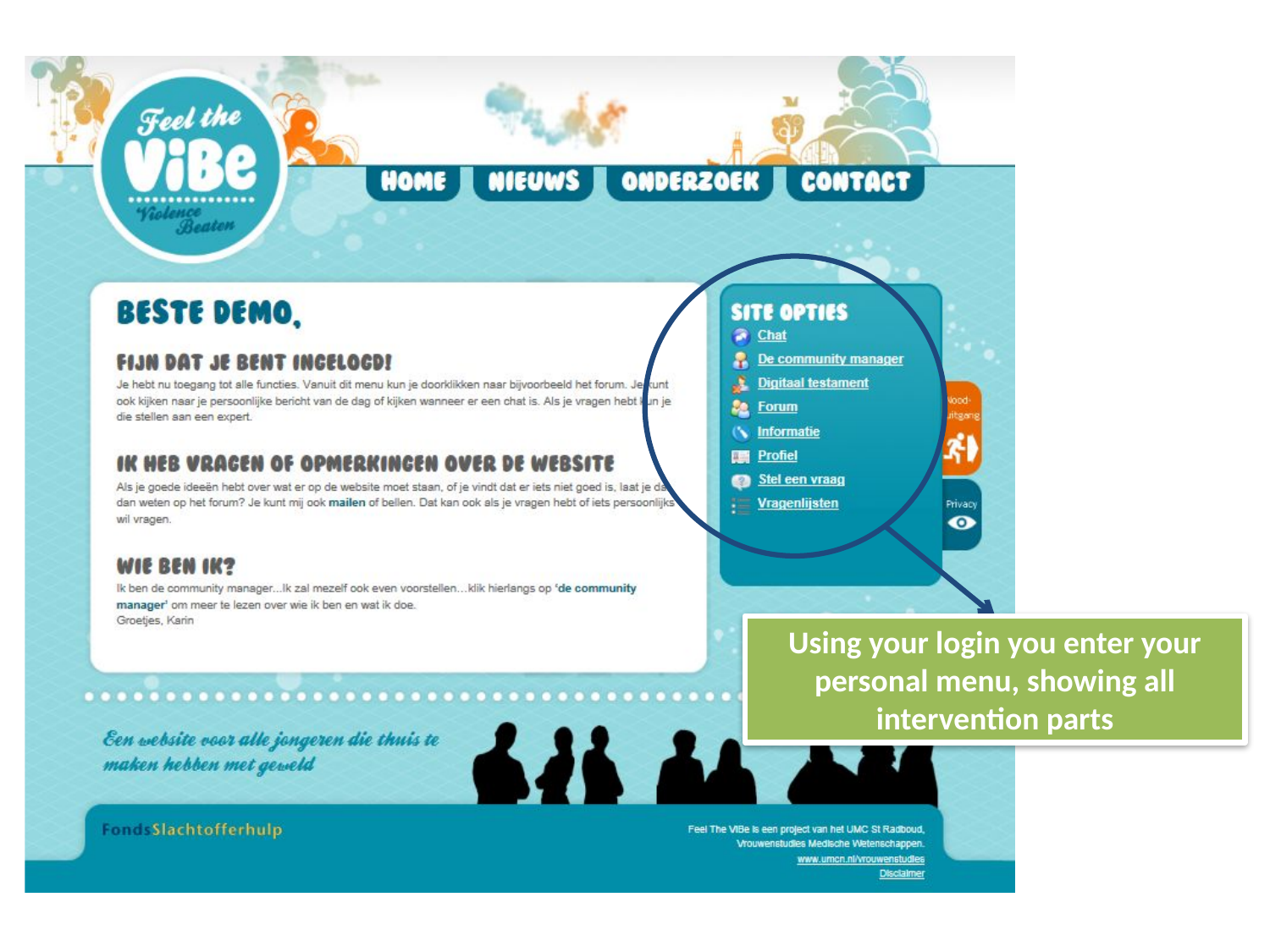

## Slide 8
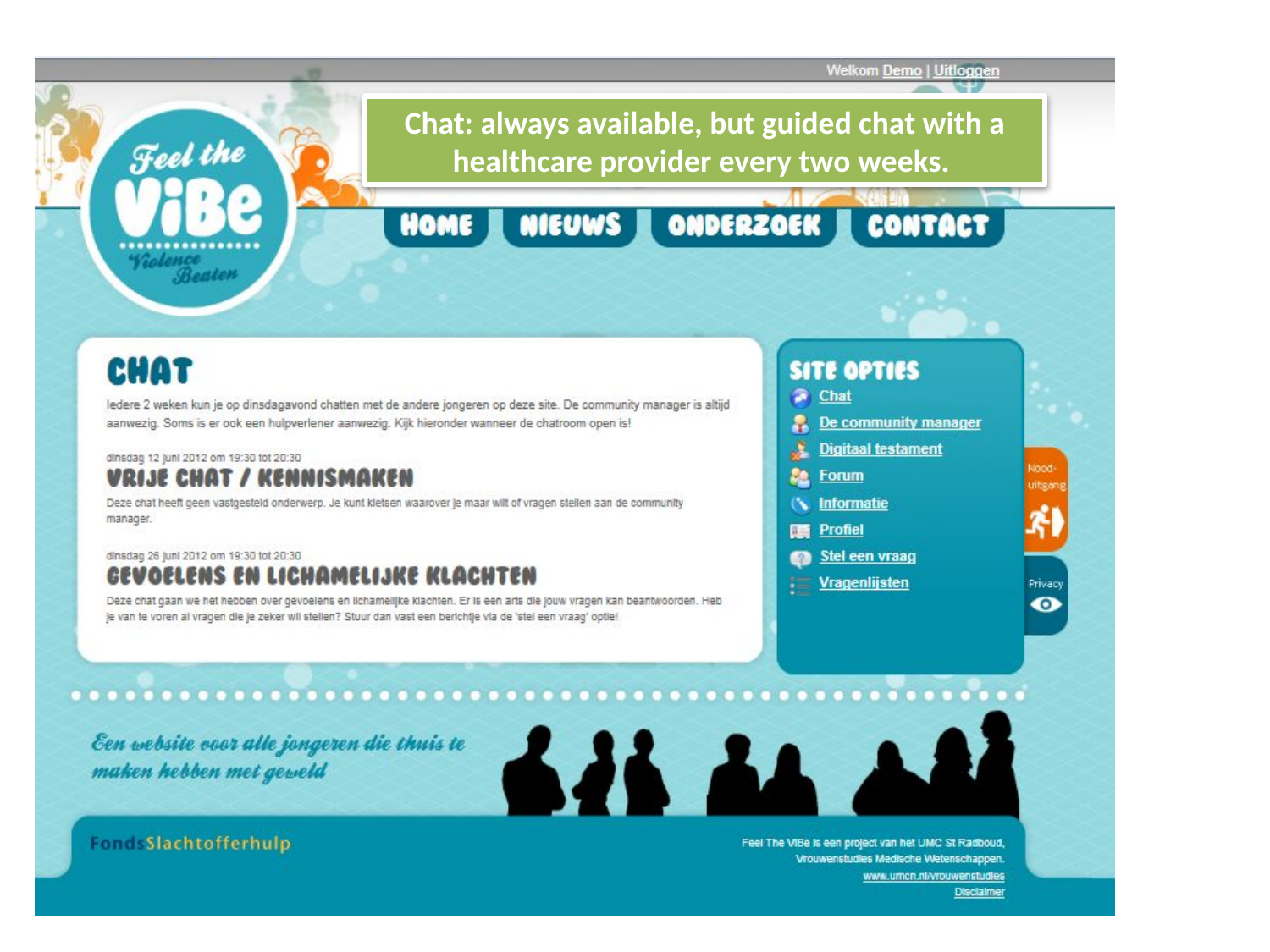

## Slide 9
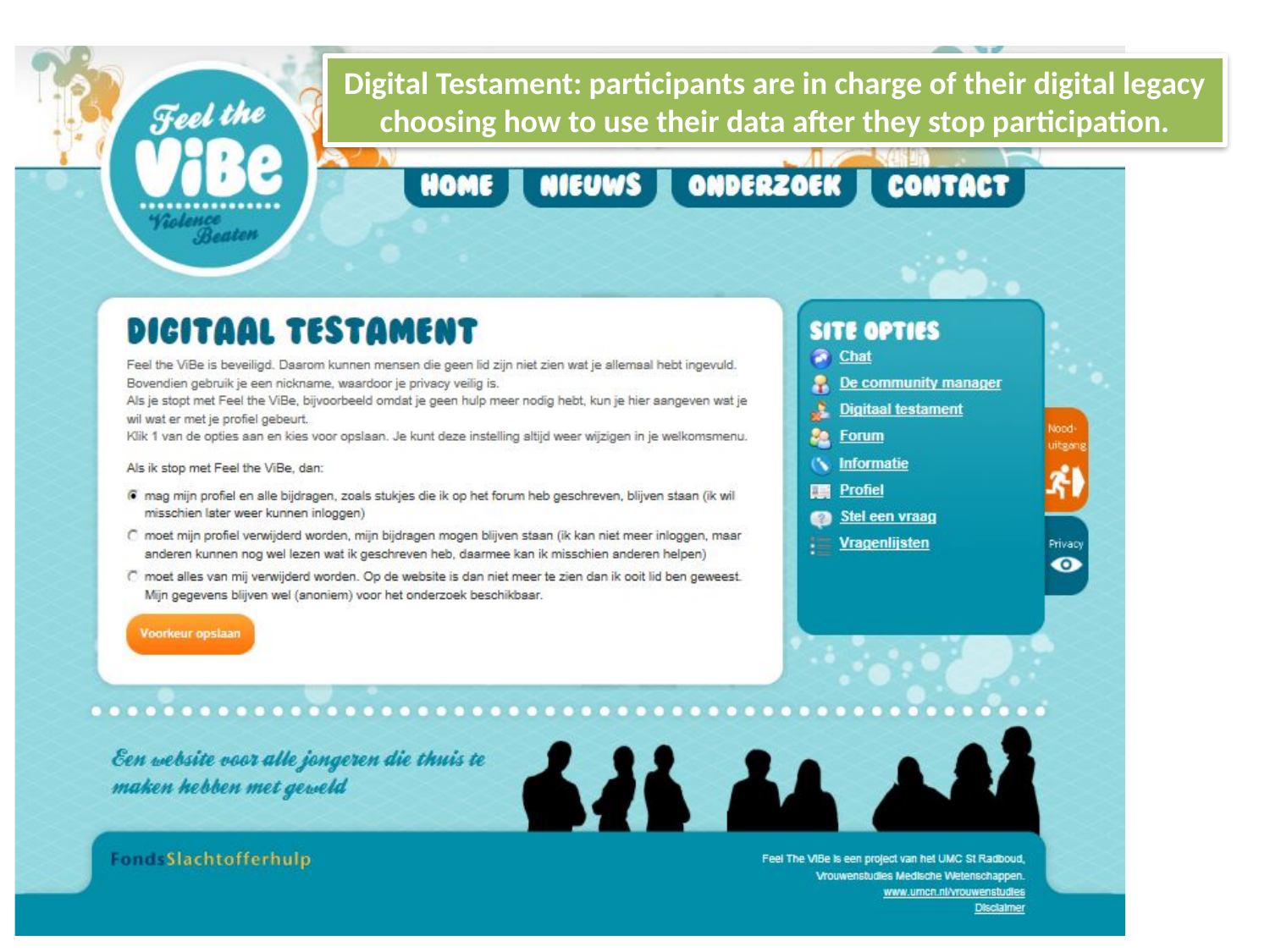

## Slide 10
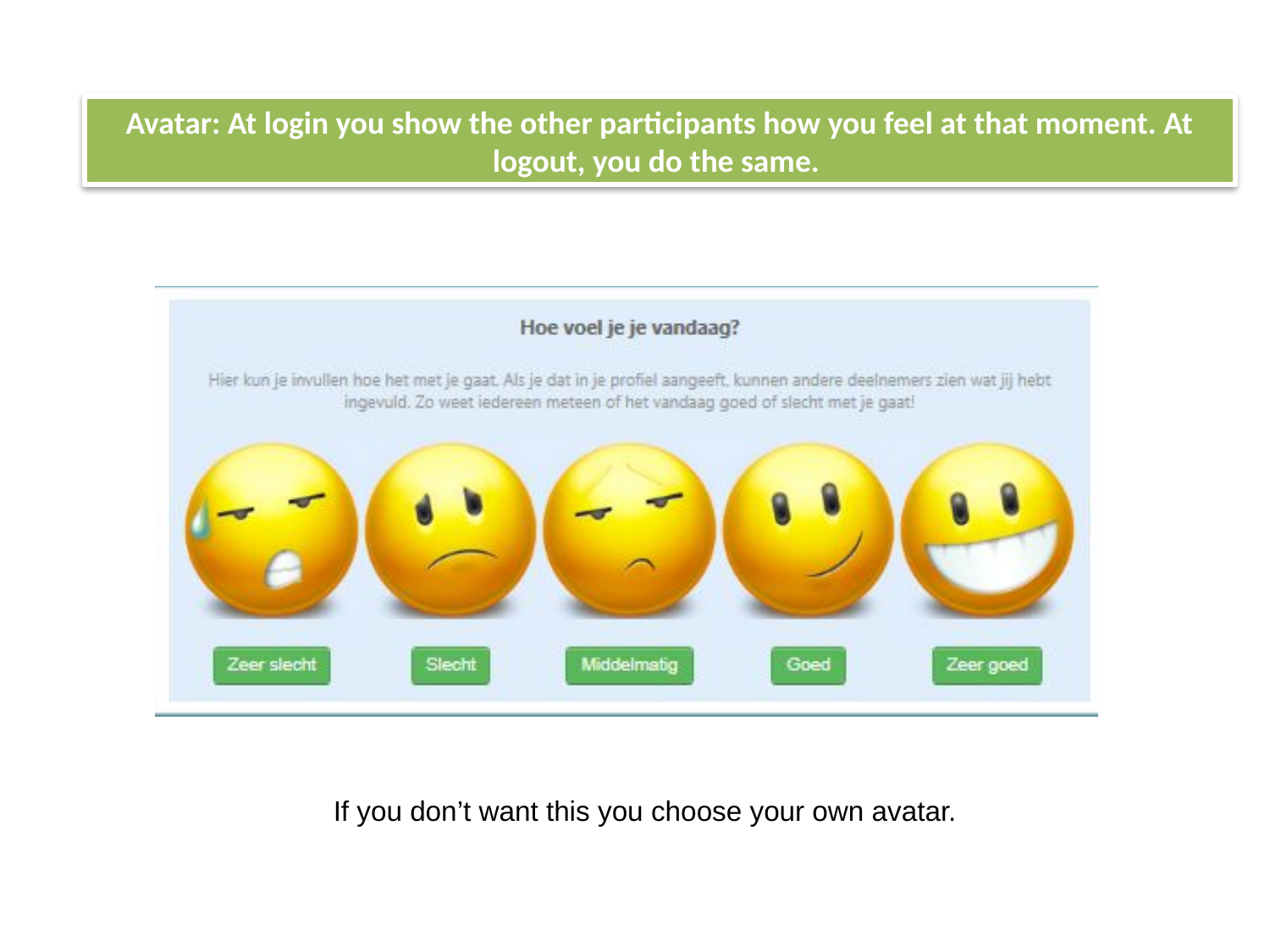

## Slide 11
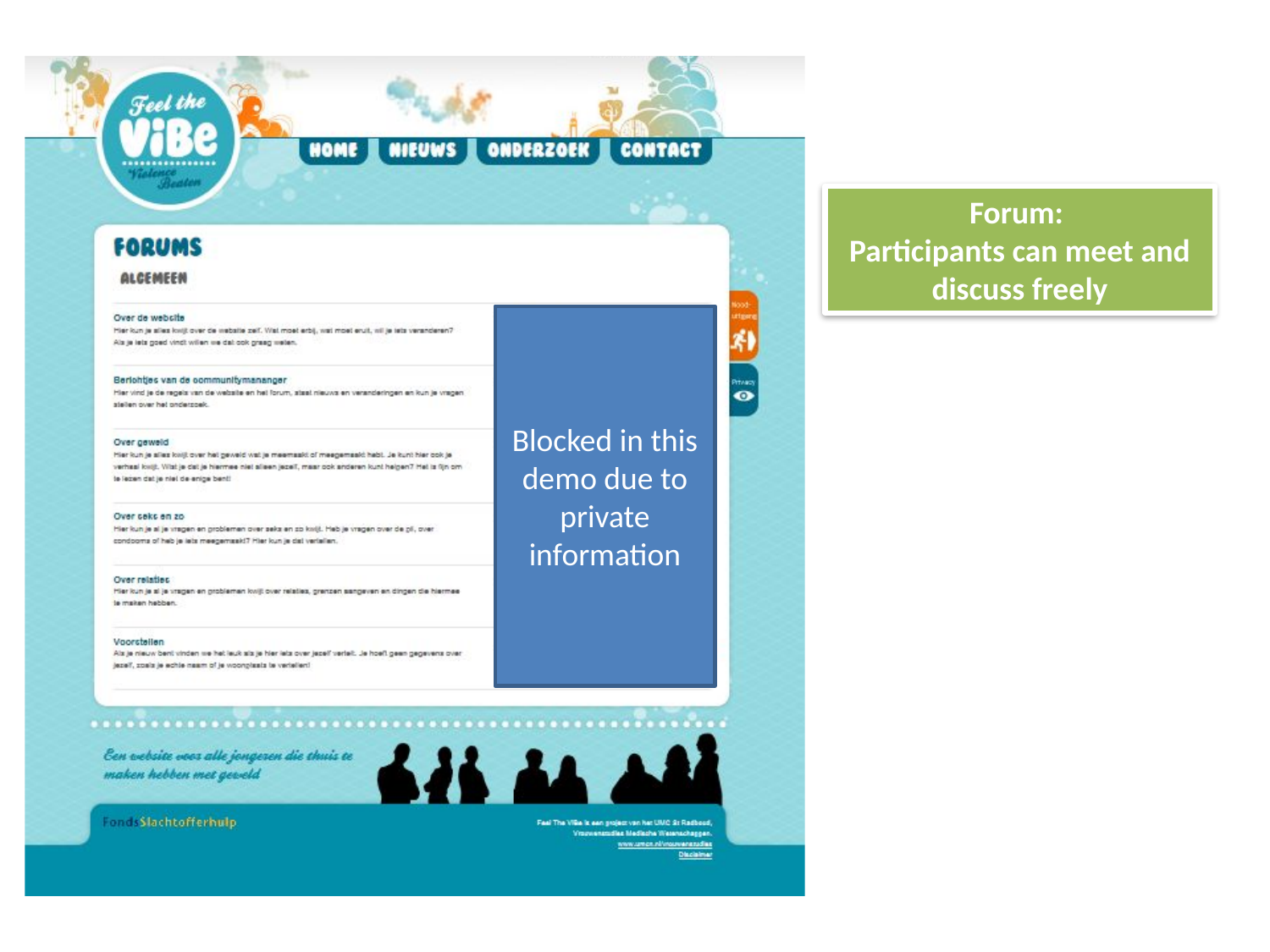

## Slide 12
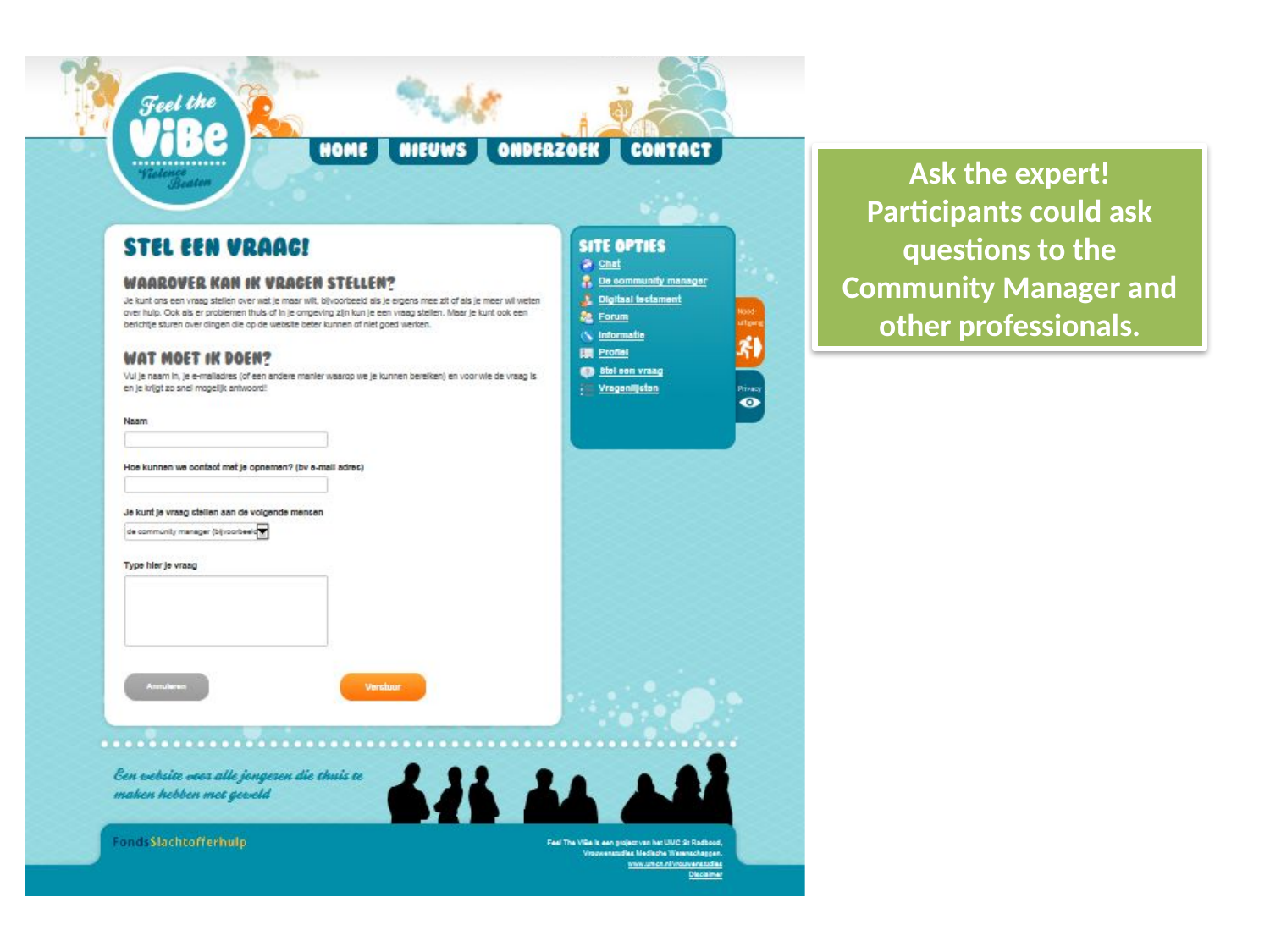

## Slide 13
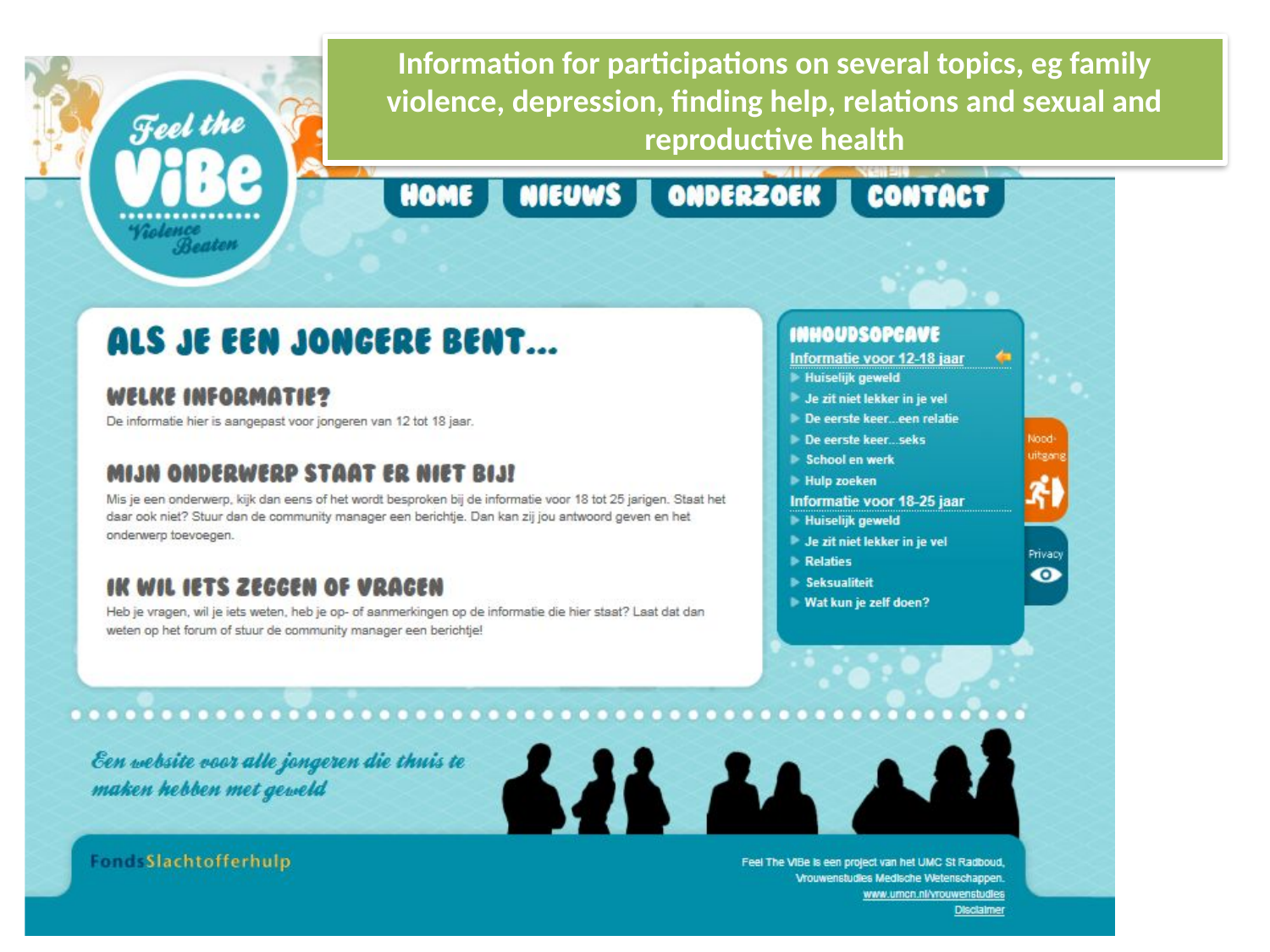

## Slide 14
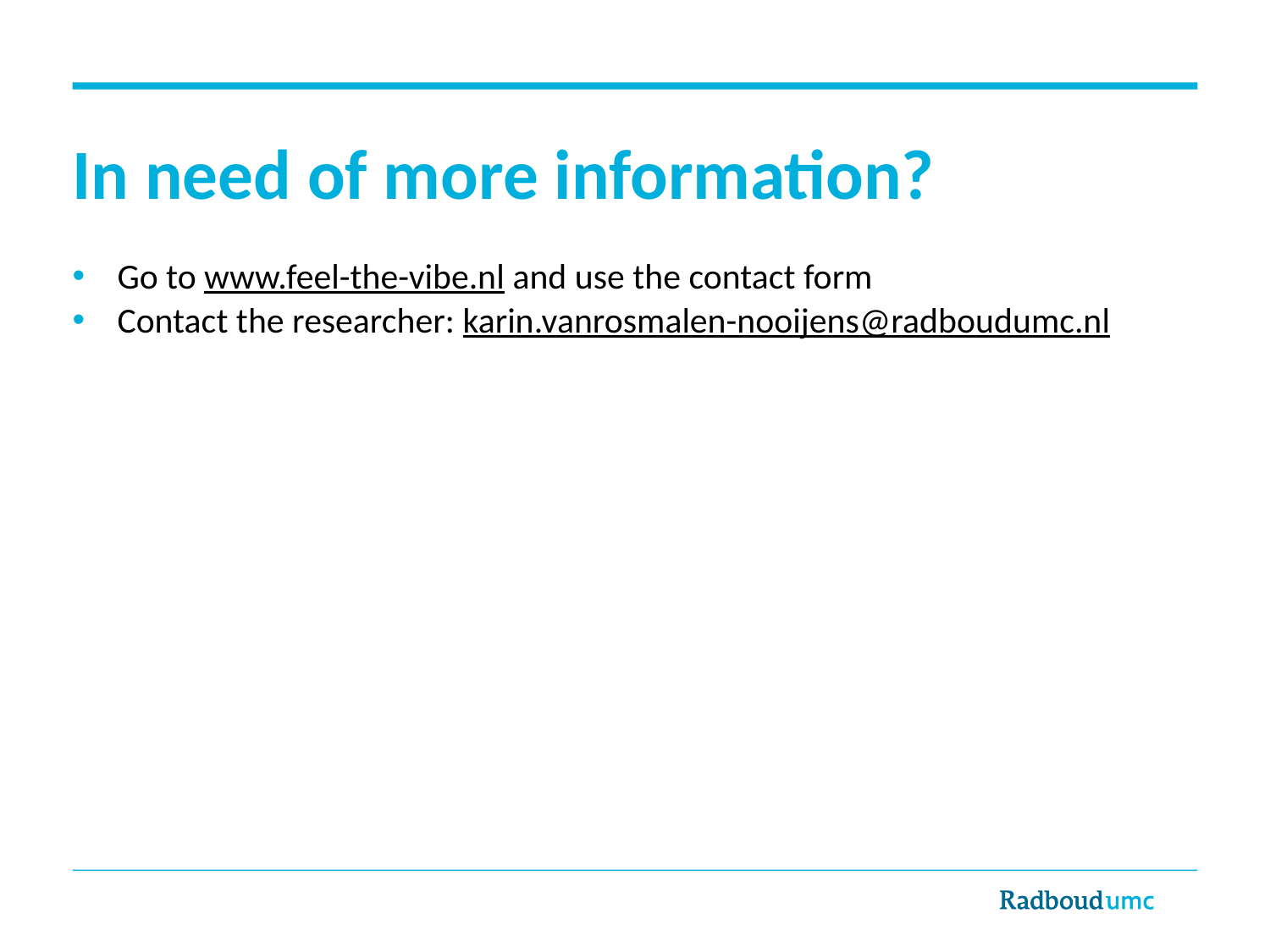

Supplement: Multimedia Appendix 1 [file jmir_v19i6e204_app1.ppsx]
